# Fantastic databases and where to find them: Web applications for researchers in a rush

**DOI:** 10.1590/1678-4685-GMB-2020-0203

**Published:** 2021-04-02

**Authors:** Gerda Cristal Villalba, Ursula Matte

**Affiliations:** 1 Hospital de Clínicas de Porto Alegre, Laboratório de células, tecidos e genes, Porto Alegre, RS, Brazil.; 2 Universidade Federal do Rio Grande do Sul, Programa de Pós-Graduação em Genética e Biologia Molecular, Porto Alegre, RS, Brazil.; 3 Hospital de Clínicas de Porto Alegre, Bioinformatics Core, Porto Alegre, RS, Brazil.; 4 Universidade Federal do Rio Grande do Sul, Departamento de Genética, Porto Alegre, RS, Brazil.

**Keywords:** Human databases, bioinformatics tools, web application, data mining, big data

## Abstract

Public databases are essential to the development of multi-omics resources. The amount of data created by biological technologies needs a systematic and organized form of storage, that can quickly be accessed, and managed. This is the objective of a biological database. Here, we present an overview of human databases with web applications. The databases and tools allow the search of biological sequences, genes and genomes, gene expression patterns, epigenetic variation, protein-protein interactions, variant frequency, regulatory elements, and comparative analysis between human and model organisms. Our goal is to provide an opportunity for exploring large datasets and analyzing the data for users with little or no programming skills. Public user-friendly web-based databases facilitate data mining and the search for information applicable to healthcare professionals. Besides, biological databases are essential to improve biomedical search sensitivity and efficiency and merge multiple datasets needed to share data and build global initiatives for the diagnosis, prognosis, and discovery of new treatments for genetic diseases. To show the databases at work, we present a a case study using *ACE2* as example of a gene to be investigated. The analysis and the complete list of databases is available in the following website <https://kur1sutaru.github.io/fantastic_databases_and_where_to_find_them/>.

## Introduction

The advent of sequencing technologies has motivated the development of public databases initiatives. Recently, there has been an exponential growth in generation of biological data, and these require information technology tools to store, organize and analyze biological data that is available in the form of raw data, annotated sequences, tables, and other archive files generated by omics technologies (Toomula *et al.*, 2012).

**Figure 1 -:**
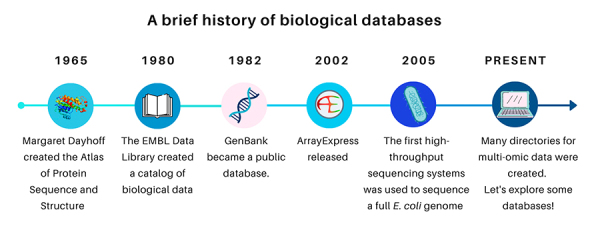
A brief history of the biological databases.

The first reported biological database was a protein sequence database developed by Margaret Dayhoff in 1965. She also created the first substitution matrix for point accepted mutations (PAM) and the one-letter code for amino acids (Strasser, 2012). In the early 1980s, the EMBL Data Library (currently European Nucleotide Archive, <https://www.ebi.ac.uk/ena>) created a catalog of published biological data. A timeline with some historical facts about biological databases are provided in Figure 1.

A biological database is a collection of data organized in systematic contents that can quickly be accessed, managed, and updated (Toomula *et al.*, 2012). Biological databases usually use relational management frameworks and the Standard Query Language (SQL), which allows data definition as well as data manipulation statements (Kriegel and Schonauer, 2004).

According to the level of data curation, biological databases are divided into primary and secondary databases. Primary databases store experimental data from nucleotide sequence, protein sequence, or molecular structure. Secondary databases comprise data from the results of analyzing primary data and has become a reference library for just about any gene or gene product that has been investigated by the research community (Selzer et al., 2008; Zou et al., 2015).

The National Center for Biotechnology Information (NCBI, <https://www.ncbi.nlm.nih.gov/>) classifies the biological databases in Comprehensive and Specialized. Comprehensive databases store data from many organisms and many different types of sequences, for example, nucleotide, protein, and genomes. Specialized databases contain data from specific organisms (e.g., humans or mice), with functional or sequence information, and data generated by specific sequencing technologies.

Here we present an overview of human databases with web applications. The databases and tools allow to search biological sequences, genes and genomes, gene expression patterns, epigenetic variation, protein-protein interactions, variant frequency, regulatory elements, and comparative analysis between human and model organisms. Our goal is to provide an overview of available tools for exploring large datasets and analyzing the data for users with little or no programming skills.

## Methods

We reviewed the databases from two platforms of multi-omic data: Biotools: Bioinformatics Tools and Services Discovery Portal (Ison et al., 2013, <https://bio.tools/>) and OMICtools: an informative directory for multi-omic data analysis (Henry et al., 2014, <https://omictools.com/>). We revised the tools from 15 March 2020 to 15 April 2020.

In the OMICtools portal, we used the keywords “human resources”, in the “Databases” tab, sorted by “A to Z” and Taxonomy: “*Homo sapiens*”. Subsequently, we discarded the results found that were of non-human species. In the Biotools portal, we used the keywords “human” and “Web application”, the categories were found in the description of the filtering tools. We discarded the tools with the ‘Temporarily unavailable’ flag.

## Results

### The Newt Scamander and its magic creatures

**Figure 2 -:**
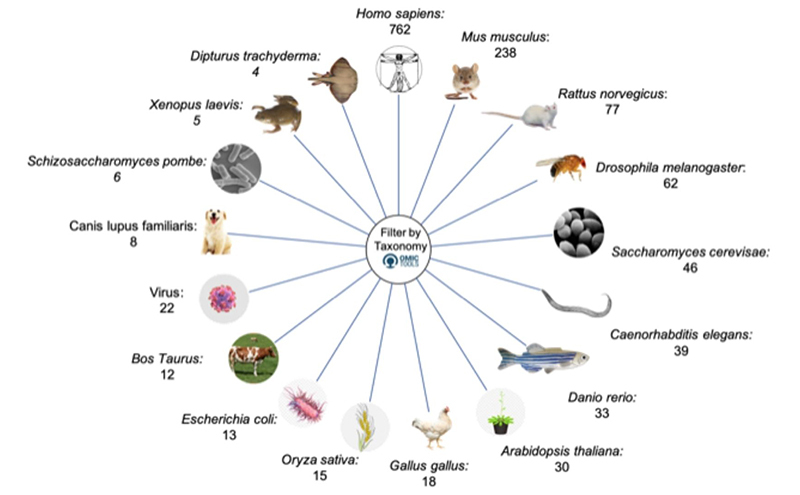
Omictools databases and tools ordered by species. In this work, we used only the tools and databases for humans.

The Omictools portal includes more than 4400 tools, classified by software, protocols, datasets, operation system, distribution when web user interface was selected. When filtered by the organisms represented in the tools, we obtained 1390 tools (Figure 2). When we chose only human results, we obtained 762 tools, divided in to Genomic Databases (91), Gene expression (81), miRNA (50), Protein-Protein Interaction (45), Rare/low frequency variation (28), Variant-disease association (28), Disease-specific variation (24), Proteome (23), LncRNA (17), miRNA target (17), Transcription Factor (17), DNA methylation (16), Metabolic network (15), Alternative Splicing (14), Cis-regulatory DNA element (12), Genetic association (12), Sequence databases (12), Comparative Genome (10), Enzyme databases (10), Gene regulatory network (10). Some tools were found in more than two of the classifications above. Besides, some of the tools were with the sites under maintenance or unavailable. For human databases, we revised 505 tools, detailed in Tables S1-S9.

**Figure 3 -:**
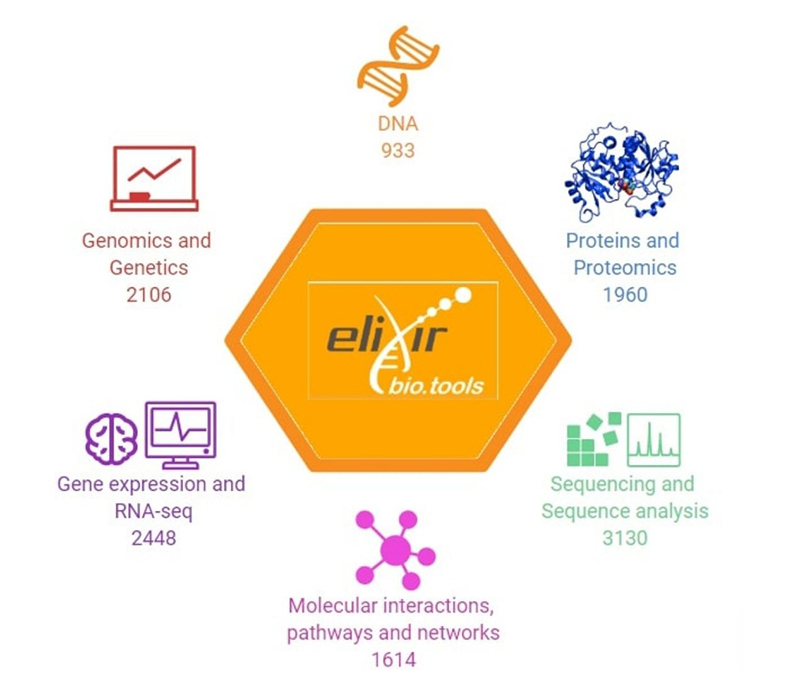
Bio.tools databases ordered by type.

In the Biotools portal, we found a total of 17,234 tools, divided into eight popular terms: Genetics, Proteins, Nucleic acids, Sequence analysis, Structure analysis, Omics, Virology and vaccine design, and Other. In order to facilitate the count of tools, we subdivided the classification in six terms presented in Figure 3. Filtered by human tools, we found 838 tools, and restricting to only “human Web Applications”, we found 235 tools. When excluded the unavailable tools, we obtained 178 tools listed in Tables S1-S9.

**Figure 4 -:**
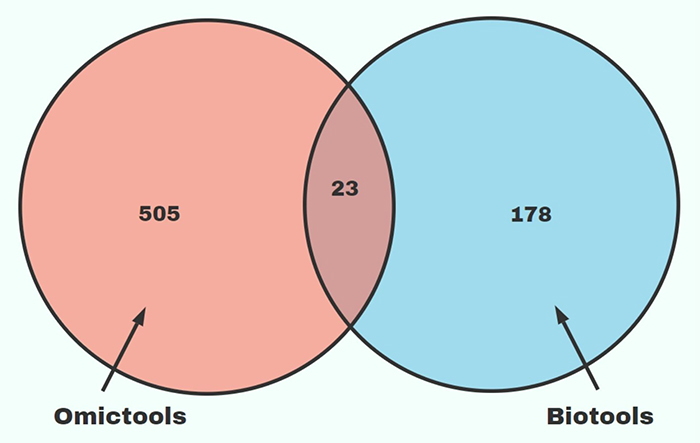
Venn diagram of the resources used for the review of databases.

There are only 23 tools present in the two portals: Clinvar, COSMIC, dbGAP, dbMAE, dbSNP, DisGeNET, DO, GENCODE, GeneCards, GIANT, GTEx, Gencode, GeneCards, GTEx, HumanMine, HuPho, InvFEST, LOVD, OMIM, Pickle, ProteomicsDB, Pseudomap, Varsome (Figure 4). In the next topic, we provide a brief description of the functionality and types of analysis that we can perform with the tools presented in this work. To facilitate, we grouped the tools into twelve general terms.

### Databases and resources highlights

To illustrate the use of the databases reviewed here, we provided a Case Study with omic analysis using the *ACE2* gene, the receptor of coronavirus SARS-Cov-2. The Case Study is available on <https://kur1sutaru.github.io/fantastic_databases_and_where_to_find_them/>.

### Alternative splicing

In summary, we found 24 tools for splice site analysis, to analyze the effect of alternative splicing on protein interaction and network through alteration of protein structure, and to predict a splicing consequence of an SNV at intron positions in the human genome (Table S1). Other databases search for constitutively and alternatively spliced introns and exons in humans and compare with other species. They also predict occurrences of alternative-splicing (AS) modes in the human genome, including exon skipping, 5’-alternative splicing, 3’-alternative splicing and intron retention. Some databases provide a curated catalog of Alternative Splicing sites and gene transcripts, and to assign the function to the human protein-coding splice variants (PCSVs). Most of the results are obtained in molecular studies and based on Machine Learning predictions. Usually, the input is the Gene ID or Symbol, or a Fasta file containing the interest sequence. Some tools present the results in the browser with graphical reports and results are available for download.

### Cancer databases

We found 56 tools (Table S2) and databases which provide genomic, transcriptomic, epigenomic profiles of a large group of tumor types with available tumor and normal tissue samples for comparisons, tools to predict the recognition of cancer epitopes by human T cell receptors (TCRs), literature mining of cancer-related genes in human, resources for exploring the impact of somatic mutations in human cancer, tools for inferring human and mouse gene expression patterns in various normal and cancerous tissues, and information about isoform-level expression analysis. Also, there are systematic analysis of mutations affecting cancer drug sensitivity based on individual genomic profiles from large-scale chemical screening using human cancer cell lines. Human cancer-specific microRNA- (miRNA) target interactions, protein-protein interactions (PPI), and functionally synergistic miRNA pairs are also available. aCGH data is analyzed to yield gene-specific copy numbers in different types of tumors.

Beyond this, many databases provide a compilation of cancer cell lines, driver genes, cancer metastasis, metastasis suppressor and histological characteristics, and complex queries built on the Boolean logic rules. Many tools are disease-specific, such as Human Gastric Cancer or Pediatric Cancer, for example. The user can provide Gene ID, miRNA, type of tumor, tissue, or cell line to search in the databases. The results allow us to explore, compare, and analyze all available cancer data (Clinical data, Gene Mutation, Gene Methylation, Gene Expression, Protein Phosphorylation, Copy Number Alteration, and so on). Outputs include barplots, heatmaps, principal component analysis plots, volcano plot, and survival analysis possible to view in the browser or to download.

### Comparative databases

We found 31 tools and databases on this topic (Table S3). These tools give a comparative phenome-genome cross-species identification of genes associated with orthologous phenotypes, evolutionary analysis of genes, and comparative genomic analysis. Also comparisons between human and animal models’ genomes are available to investigate links between disease genes, experimental data dealing with antibody and T-cell epitopes studied in various animal species encompassing humans and non-human primates. Data about interacting regions (IRs) in human and mouse proteins, miRNA target sites across species, non-human primate reference transcriptomes, allow to analyze and compare human nonsynonymous SNPs (nsSNP) in protein structures, protein complexes, protein-protein interfaces, and metabolic networks. Users can browse a genome, gene or region, phenotype term, disease name, or tissue for comparison. The output may present alignment across the genomes of different animal models and gene information (species, chromosome, gene name, accession number). Other output options are alternative transcripts, phylogeny plots, and gene regulatory networks.

### Disease-specific and variant-disease association

We found 84 tools and specialized databases (Table S4) related to monogenic and complex diseases such as Alzheimer’s, Autism, mendelian genetic diseases, and variant databases, such as repositories of human mutations, and single nucleotide polymorphisms (SNV). The input, in general, is the gene ID or symbol or name of the disease. The output, in general, is a list of annotated variants with the position number (genomic or chromosomal position), and the impact of the variation (missense, nonsense, frameshift, among others). Many databases provide germline and somatic variants of any size, type or genomic location, and the possible interpretations of the clinical significance of variants for reported conditions. The user may also search for genes based on user-specific disease/phenotype terms.

### Gene expression

In this specific topic, we found 116 tools and databases with data about transcript counts, gene expression in normal and diseased tissues (Table S5). The databases comprehend data on age, apoptosis, gene expression in particular tissues or situations (such as allergy or immune response). Also, RNA-seq data from human and animal cells, single cell studies, and datasets for chromatin regulators and histone modifications are also available. The input can be a Gene ID, Pubmed ID, tissue cell or cell line, and phenotype information. The output is diverse across the databases, with tables, diagrams, heatmaps, barplot, and dotplot. Some databases present information about the gene structure, subcellular localization, primary function, cellular processes, and pathways. Many databases also inform the literature reference on the experimental data about the selected gene.

### Genomic and sequence databases

We found 217 tools (Table S6), related to post-translational (PTM) modification sites and mutations (both germline and somatic) from multiple sources, mitochondrial sequences coming from ancient DNA samples (aDNA), disease-specific genomic data, genomic histone marks, chromatin states and motifs, chromosome annotation, evolutionary relationship of human proteins and animal models, analyses of allelic imbalance in clonal cell populations based on sequence polymorphisms. General information on genes, such as orthologs and paralogs, exon, intron and UTRs, gene classification, transcript sequences, protein sequences, mutations and SNPs, transcript cluster or selected publications can also be found. The general input is a Gene Symbol, Chromosomal position or region. The output is presented in browser visualization, tables with the genomic sequence of the interest, gene report, and heatmaps with pathogenicity of the mutations found in a sequence of the gene or region of interest.

### LncRNA and miRNA databases

We found 73 tools (Table S7) related to miRNA and long non-conding RNAs (lncRNAs) in different organisms and pathogenic conditions. Of special interest may be data on putative antagomirs-miRNA heterodimers, protein-protein interactions (PPI) and functionally synergistic miRNA pairs (including transcription factors). The input of the search can be a Gene symbol, miRNA or LncRNA ID or target (hsa-let-7a, for example), Fasta file of the region of interest, or chromosomal location (any position in hg38 version, for example). In the case of LncRNA, the output is a table with the basic information of sequence, strand, class (antisense, for example), and conservation across species, with or without prediction scores. For miRNA, the output show information about the seed region, conservation score, accessibility, and secondary structures, minimum free energy estimation, correlations about expression, and start/end of the miRNA sequences. In disease-specific databases, the table may present the disease name, information about causality, and related literature.

### Metabolic and enzyme databases

We found 24 tools (Table 1) involving databases with information about potential cleavage sites in datasets of all human proteins collected in Uniprot and their orthologs, allowing for tracing of cleavage motif conservation, bioactive molecules, human metabolites, natural products, patented agents and other molecules. Databases are organized as disease-specific, tissue-specific, organelle-related or by protein families. Epigenetic enzymes and chemical modulators focused on epigenetic therapeutics, as well as functional databases including information from cells and tissues at a variety of physiological conditions, are also available. The input of the analyses is either a GO term, gene symbol or name, chromosomal location, metabolite or molecule name, or enzyme family name. Compartment and subsystem 2D maps, tables with literature related, enzyme domains, Uniprot ID, plots of classic or multivariate ROC curves, and images illustrating predicted small molecules are present in the output.

**Table 1 -:** Metabolic and enzyme databases.

Name	URL	Brief description	Download of Data	Current status
1-CMDb	http://slsdb.manipal.edu/ocm/	Multi omics associated with one carbon metabolism	Yes	Online
CaspDB	http://caspdb.sanfordburnham.org	Cleavage sites in proteins collected in Uniprot and their orthologs	No	Offline
CFam	http://bidd2.nus.edu.sg/cfam	Cluster drugs, bioactive molecules, human metabolites, natural products, patented agents and other molecules	No	Offline
CIDeR*	http://mips.helmholtz-muenchen.de/cider/	Information from neurological and metabolic diseases	Yes	Online
DESTAF	https://www.cbrc.kaust.edu.sa/destaf/	Metabolism and toxins in diseases and tissues	Yes	Online
dkNET	https://dknet.org//	Integrated data of Diabetes and Digestive and Kidney Diseases	Yes	Online
HEMD	http://mdl.shsmu.edu.cn/HEMD/	Human epigenetic enzymes and chemical modulators	Yes	Online
HEPATONET1	http://www.ebi.ac.uk/biomodels-main/MODEL1009150000	Genome-scale metabolic network of human hepatocytes	No	Online
HMA	https://metabolicatlas.org/	Comprehensive human metabolic information as models	Yes	Online
HumanCyc	https://humancyc.org/	Human nutrition that associates with a set of metabolic pathways	No	Online
HMDB*	https://hmdb.ca/	Human Metabolome Database	Yes	Online
KinMap	http://www.kinhub.org/kinmap/	Interactive navigation through human kinome data	Yes	Online
KinMutRF	http://kinmut2.bioinfo.cnio.es/KinMut2	Prediction of variants in the human protein kinase superfamily	No	Offline
metabolicMine	https://www.humanmine.org/humanmine/begin.do	Metabolome profiling and model organisms	Yes	Online
MetSigDis	http://www.bio-annotation.cn/MetSigDis/	Metabolite alterations in various diseases	Yes	Offline
MSEA	https://www.metaboanalyst.ca/	Enrichment analyses for (primarily human) metabolomic studies	Yes	Online
NOPdb	http://www.lamondlab.com/NOPdb	Nucleolar proteins identified by mass spectrometry analyses	No	Offline
PeroxisomeDB	http://www.peroxisomedb.org/	Peroxisomal proteins, molecular function, metabolic pathway and disorders	Yes	Online
PhosphoPredict	http://phosphopredict.erc.monash.edu/	Predict kinase-specific phosphorylation substrates and sites in the human proteome	Yes	Online
Piphillin	http://piphillin.secondgenome.com/	Metagenomic data by Direct Inference from Human Microbiomes	Yes	Online
R spider	http://www.bioprofiling.de/gene_list.html	Pathway analysis from KEGG and Reactome	No	Online
RegenBase	http://regenbase.org/	Effect of compounds on enzyme activity and cell growth	Yes	Online
TSEM	https://hood-price.isbscience.org/research/tsem/	Tissue specific encyclopedia of metabolism and metabolic models	Yes	Online
VMH	https://vmh.life/	Human metabolism and genetics, microbial metabolism, nutrition, and diseases	Yes	Online

*Databases present in the case study.

### Methylation databases

We found 16 tools (Table 2) related to methylation patterns in aging-related diseases, normal or tumoral tissues, CpG islands and sites, and epigenome-wide studies. Information about the tissue-specific variation of methylation in the human central nervous system and matched blood samples collected from multiple donors are also available. Human cancer-specific DNA methylation, imprinting and gametogenesis-related methylation changes, and associations between RNAs and methylation databases are also available. The input generally is an Entrez ID or Gene Symbol, SNP ID (e.g., rs1001098), Chromosome Location, CpG island ID or the DNA sequence in Fasta file. Also, a target tissue can be informed. The output shows tables with gene and methylated positions, CpG location, and in some cases, the literature related to the study. Some tools show the CpG islands in the genome browser, in a graph with the content of the CpG island region, CpG sites, and the DNA sequence.

**Table 2 -:** Methylation databases.

Name	URL	Brief description	Download of Data	Current status
ANCOGeneDB	https://bioinfo.uth.edu/ancogenedb/	Epigenomic, enhancers, and expression quantitative trait loci	Yes	Online
BECon*	https://redgar598.shinyapps.io/BECon/	Interpreting methylation findings from blood in the context of brain	Yes	Online
CMS	http://cbbiweb.uthscsa.edu/KMethylomes/	Analytic functions for cancer methylome datasets	No	Offline
DBCAT	http://dbcat.cgm.ntu.edu.tw/	Methylation profiles of DNA alteration in human cancer	Yes	Online
DiseaseMeth*	http://bio-bigdata.hrbmu.edu.cn/diseasemeth/	Aberrant methylomes of human diseases	No	Online
GED	http://gametsepi.nwsuaflmz.com/	Epigenetic modification of gametogenesis in mammals	Yes	Online
Geneimprint	http://www.geneimprint.com/site/home	Gene imprinting and which allele is expressed	Yes	Online
Lnc2Meth	http://bio-bigdata.hrbmu.edu.cn/Lnc2Meth/	Informs about RNAs and DNA methylation of transcripts	Yes	Online
MeInfoText	http://bws.iis.sinica.edu.tw:8081/MeInfoText2/	Gene methylation and cancers, protein-protein interactions, and biological pathways	Yes	Offline
MethHC	http://methhc.mbc.nctu.edu.tw/	Focuses on aberrant methylomes of human diseases	No	Offline
MethylomeDB	http://epigenomics.columbia.edu/methylomedb/index.html	DNA methylation profiles for human and mouse brains	Yes	Offline
mPod	www.genome.org	Genome-wide tissue-specific DNA methylation profiles	No	Online
PhenoScanner	http://www.phenoscanner.medschl.cam.ac.uk/	Methylation and human genotype-phenotype associations	Yes	Online
ROADMAP	https://egg2.wustl.edu/roadmap/web_portal/index.html	Epigenetic modifications and mRNA expression of human cell types and tissues	Yes	Online
TCGA	https://www.cancer.gov/	Cancer methylation and expression	Yes	Online
TSGene	http://bioinfo.mc.vanderbilt.edu/TSGene/	Methylation status of tumor suppressor genes	No	Offline

*Databases present in the case study.

### Proteome and protein-protein interaction

In this topic, we found 97 databases (Table S8) that investigate the behavior of protein subunits in known complexes by comparing their abundance profiles across cell types, also tools for the identification of protein hydroxylation sites, to classify and score human coding variants based on the probability to damage their protein-related function. Databases of molecular-level putative protein-drug interactions, explorable and interactive human proteome database including MS/MS data, databases of protein interaction information pre-computed from existing structural and experimental data were also found. Fasta protein sequences, or Uniprot and Gene ID, are the input for these tools. Interactions between metabolites and molecules, small compounds, and enzyme’s families and characterization are the output.

### Regulatory elements

The 29 databases (Table 3) allow to visualize modified ribosomal nucleotides of human and several major model organisms, present resources to the identification of transcription factors, functional elements, cis-regulatory elements, interferon regulated genes, large intergenic non-coding RNAs (lincRNAs) and miRNA regulatory cascades in human diseases, Triplex Target DNA Site (TSS) with genomic regulatory sequences and signals, and RNA binding elements. The data is either from text-mining-assisted workflow, chromatin immunoprecipitation (ChIP), high-throughput datasets, Genome-Wide Association Studies (GWAS), next-generation sequencing techniques and/or predicted by computational models with annotations obtained by expert review of the scientific literature. Gene names, accession numbers, Fasta sequences, ligand ID (e.g., G4L0021), ligand name (e.g., TMPyP4), ligand activity or binding properties (e.g., Cytotoxicity), author name of ligand related literature, Ensembl ID List, tissue or cell type are examples of the possible inputs. Databases for Triplex target DNA sites provide specific search criteria, such as percent guanine content and pyrimidine interruption. The result shows a list of genes, tables, venn diagrams, scatter plot, position weight matrix for a selected motif, navigation across the motifs, and heatmaps with the target elements and biosamples.

**Table 3 -:** Regulatory databases.

Name	URL	Brief description	Download of Data	Current status
ChIPSummitDB	http://summit.med.unideb.hu/summitdb/index.php	ChIP-seq-based data of transcription factor binding sites and the topological arrangements of the proteins	Yes	Online
CREME	https://creme.dcode.org/	Cis-regulatory module explorer for the human genome	Yes	Online
CRUNCH	http://crunch.unibas.ch/crunch/	ChIP-seq data analysis	Yes	Online
FirstEF	http://rulai.cshl.org/tools/FirstEF/	First Exon Finder (FirstEF) is a 5’ terminal exon and promoter prediction program	Yes	Online
GlycoViewer	http://www.glycoviewer.babs.unsw.edu.au/	Visualisation tool for representing a set of glycan structures	Yes	Online
HERVd	https://herv.img.cas.cz/	Human endogenous retroviruses database	No	Online
HumCFS	https://webs.iiitd.edu.in/raghava/humcfs/index.html	Human chromosomal fragile sites data	No	Online
Interferome*	http://interferome.its.monash.edu.au/interferome/home.jspx	Contains type I, II and III interferon (IFN) regulated genes	No	Online
JASPAR	http://jaspar.genereg.net/	The high-quality transcription factor binding profile database	Yes	Online
MANTA	http://manta.cmmt.ubc.ca/manta2/upload	Maps of transcription factor binding sites	Yes	Online
MAPPER	http://genome.ufl.edu/mapperdb	Multi-genome analysis of positions and patterns of elements of regulation	No	Offline
MEME Suite	http://meme-suite.org/	DNA motifs, transcription factor binding sites or protein domain	Yes	Online
MET	http://veda.cs.uiuc.edu/MET/	The motif enrichment tool identifies significantly associated sets of genes that share a regulatory motif	Yes	Online
microDoR	http://reprod.njmu.edu.cn/cgi-bin/microdor/index.py	Predict Human miRNA-mediated gene silencing	Yes	Online
OsteoporosAtlas	http://biokb.ncpsb.org/osteoporosis/index.php	Regulatory sequences in osteoporosis-related genes	Yes	Online
PReMod	http://genomequebec.mcgill.ca/PReMod	Predict transcriptional regulatory modules of human genome	Yes	Online
pseudoMap	http://pseudomap.mbc.nctu.edu.tw/php/index.php	Gathers information about transcribed pseudogenes	No	Offline
SM-TF	http://zoulab.dalton.missouri.edu/SM-TF/	Database of small molecule-transcription factor complexes	Yes	Online
SNP@lincTFBS	http://210.46.85.180:8080/SNP_linc_tfbs/	SNPs in potential TFBSs of human Large intergenic non-coding RNAs (lincRNAs)	Yes	Offline
TcoF-DB	https://tools.sschmeier.com/tcof/home/	Human transcription co-factors and transcription factor interacting proteins	No	Online
TFBSbank	http://tfbsbank.co.uk/	Chip-seq data of 585 transcription factors in 5 species	Yes	Online
TFCat	http://www.tfcat.ca/	Curated catalog of mouse and human transcription factors	No	Offline
TFClass	http://tfclass.bioinf.med.uni-goettingen.de/	Eukaryotic TFs according to their DNA-binding domains	No	Online
TFCONES	http://tfcones.fugu-sg.org/	Transcription factor genes and conserved noncoding elements	Yes	Online
TFM-Explorer	https://bioinfo.lifl.fr/TFM/	Putative TFBS within a set of upstream regulatory sequences for a given set of genes	Yes	Online
TMREC	http://www.jianglab.cn/TMREC/	TF and miRNA regulatorY cascades in human diseases	Yes	Online
TRANSFAC	http://genexplain.com/transfac/	Eukaryotic TF, their experimentally-proven binding sites, consensus binding sequences and regulated genes	No	Online
TTSMI database*	http://ttsmi.bii.a-star.edu.sg/	Triplex target DNA site mapping and integration database	No	Online

*Databases present in the case study.

### Other specialized databases

We found 64 databases (Table S9) related to immunogenetics and antigen tumor receptors, genome-wide studies, rare diseases, cell culture, experimental design, genetic traits, and human copy number variations. The immunogenetic databases focus in cytokine receptors, their ligands, their involvement in diseases and their use in clinical treatments, human Major Histocompatibility Complex (MHC) genes, peptides, predictions, and proteins about human leukocyte antigen (HLA) I and HLA II-restricted peptides, *in silico* prediction of epitopes, tumor T cell antigens and literature about immunoproteins. Genome-wide databases compiled various public resources dealing with summary-level genome-wide association studies (GWAS) results. Rare disease databases comprehend specialized databases in rare diseases, such as the DECHIPER database, LungMap, and MARRVEL. Cell line databases integrated molecular authentication and identification tools for human and animal cell lines available from some of the main European cell banks, with curated literature. Experimental design databases contain experimental data and results from studies that have investigated the interaction of genotype and phenotype in humans, and CRISPR knockout libraries to target custom subsets of genes in the human or mouse genome. Genetic traits databases contain information about the global distribution of genetic traits. Copy number variations databases provide curated reference databases and bioinformatics resources targeting copy number profiling data in human diseases, especially in cancer. In general, the input is the Gene ID, DNA sequence in a Fasta format, organism, antigen name, haplotype, disease name, or ontology. For immunogenetic databases, the output is epitope sequences with scores, a list of antigens, assays, and receptors with the respective literature reference. Genome-wide databases present tables with the GWAS summarization of the study, SNP id, hits, allelic p-value, genotypic p-value, and Manhattan plot with the significant SNPs. Experimental design databases contain lists and tables with information about disease profiles and conditions with curated literature reviews. Genetic traits databases provide lists with SNP-trait associations in different human populations, and functional regions overlapping with SNPs in high linkage disequilibrium. Also, demographic information such as Country and Ethnicity is displayed within other GWAS information according to GWAS catalog guidelines.

### Future and Perspectives

Biological databases are essential to provide information on normal and disease conditions, to search for information about DNA sequences, RNA, protein, and all possible data from different species and animal models. Public user-friendly web-based databases facilitate data mining and the search for information applicable to healthcare professionals. Besides, biological databases are essential to improve biomedical search sensitivity and efficiency and merge multiple datasets needed to share data and build global initiatives for the diagnosis, prognosis, and discovery of new treatments for genetic diseases. Gathering information about primary and secondary biological databases in a single review centralizes the search for recent and easy-to-use bioinformatics tools that can help to address some of the challenges in (Big) Data-driven research.

## Supplementary material

The following online material is available for this article:

Table S1 - Alternative splicing databases.

Table S2 - Cancer databases.

Table S3 - Comparative databases.

Table S4 - Disease-specific and variant-disease association.

Table S5 - Gene expression databases.

Table S6 - Genomic and sequence databases.

Table S7 - LncRNA and miRNA databases.

Table S8 - Proteome and protein-protein interaction databases.

Table S9 - Other databases.
