# Supplementary material for: Fantastic databases and where to find them: Web applications for researchers in a rush
Source: Genet Mol Biol. 2021 Apr 2;44(2):e20200203. doi: 10.1590/1678-4685-GMB-2020-0203 (PMC8022358; doi:10.1590/1678-4685-GMB-2020-0203)
Supplement: Table S1 - [file 1415-4757-GMB-44-2-e20200203-s1.pdf]

Genetics and Molecular Biology
https://doi.org/10.1590/1678-4685-GMB-2020-0203

Supplementary Material to "Fantastic Databases and where to find them: Web applications for researchers in a rush"

Table S1 - Alternative splicing databases.

Name	URL	Brief description	Download of Data	Current status
AltExtron	http://bioinformatics.org.au/tools/altExtron/	Constitutively and alternatively spliced introns and exons, for conservation in mouse.	Yes	Online
AS-ALPS	http://as-alps.nagahama-i-bio.ac.jp/index.php	Alternative splicing-induced alteration of protein structure	Yes	Online
ASpedia	http://combio.snu.ac.kr/aspedia/index.html	Alternative splicing encyclopedia with genomic annotation	Yes	Online
ASPicDB*	http://srv00.recas.ba.infn.it/ASPicDB/	Alternative splicing pattern of human genes, and functional annotation of predicted isoforms	Yes	Online
BrainRNA-seq	https://www.brainrnaseq.org/	Alternative splicing events in cell types	Yes	Online
DBASS	http://www.dbass.org.uk/	Splice-site prediction, and definition of auxiliary splicing signals	Yes	Online
EDAS	http://www.gene-bee.msu.ru/edas/	EST-derived alternative splicing database	No	Offline
FAST DB	http://www.genosplice.com/alternative-splicing	Alternative splicing and alteration of splicing regulation	No	Online
FLJ DB	http://flj.lifesciencedb.jp/top/	Variations of transcription start site (TSS) and splicing	Yes	Online
H-DBAS	http://www.h-invitational.jp/h-dbas/	Human-transcriptome data of alternative splicing	Yes	Online
HEXEvent	http://hexevent.mmg.uci.edu/cgi-bin/HEXEvent/HEXEventWEB.cgi	Splice events based on EST information	No	Online
HOLLYWOOD	http://hollywood.mit.edu/hollywood/Login.php	Splicing patterns derived from spliced alignment of cDNAs	Yes	Online
HSF	http://www.umd.be/HSF3/index.html	Prediction of mutations in splice sites	No	Online
HYBRIDdb	http://www.primate.or.kr/hybriddb/	Identify intergenic splicing-mediated gene fusion	No	Offline
IntSplice	https://www.med.nagoya-u.ac.jp/neurogenetics/IntSplice/	Predict a splicing consequence of SNV	No	Online
IsoFunc	https://guanlab.ccmb.med.umich.edu/isofunc	A tool for assigning function to protein-coding splice variants	No	Offline
MiasDB	http://47.88.84.236/Miasdb/index.php	Molecular interactions associated with human splicing	Yes	Online
NetGene2	http://www.cbs.dtu.dk/services/NetGene2/	Splice sites in human, *C. elegans* and *A. thaliana*	No	Online
NetUTR	http://www.cbs.dtu.dk/services/NetUTR/	Prediction of splice sites in 5' UTR regions	No	Online
PALS DB	http://palsdb.ym.edu.tw/	Collection of Putative Alternative Splicing	No	Offline
SpliceInfo	http://SpliceInfo.mbc.NCTU.edu.tw/	Info of exon skipping, 5' or 3'-alternative splicing and intron retention	Yes	Offline
SplicePort	http://spliceport.cbcb.umd.edu/	Splice-site predictions for submitted sequences	No	Online
SpliceProt	http://bioinfoteam.fiocruz.br/spliceprot/index.php	Alternative spliced variants based on experimental human transcriptomic data	Yes	Online
TassDB*	http://tassdb2.leibniz-fli.de/	Search for specific genes or for genes containing tandem splice sites	Yes	Online
