# Supplementary material for: Fantastic databases and where to find them: Web applications for researchers in a rush
Source: Genet Mol Biol. 2021 Apr 2;44(2):e20200203. doi: 10.1590/1678-4685-GMB-2020-0203 (PMC8022358; doi:10.1590/1678-4685-GMB-2020-0203)
Supplement: Table S2 - [file 1415-4757-GMB-44-2-e20200203-s2.pdf]

Genetics and Molecular Biology
https://doi.org/10.1590/1678-4685-GMB-2020-0203

Supplementary Material to "Fantastic Databases and where to find them: Web applications for researchers in a rush"

Table S2 - Cancer databases.

Name	URL	Brief description	Download of Data	Current status
arrayMap	https://arraymap.org/	Resource targeting copy number profiling data	Yes	Online
CGAProject	https://mitelmandatabase.isb-cgc.org/	Gene expression profiles of normal, precancer, and cancer cells	Yes	Online
CancerNet	http://bis.zju.edu.cn/CancerNet/	Contains cancer-specific miRNA and protein-protein interactions	Yes	Online
CanGEM	http://www.cangem.org/	Clinical information, transcriptome, genome, and aCGH data	No	Offline
CanProVar	http://canprovar.zhang-lab.org/index.php	Conservation, protein expression, and domains in cancer data	Yes	Online
CARGO	http://cargo2.bioinfo.cnio.es/	Visualize 3D SNPs, and view summarized gene annotation	Yes	Online
CaSNP	http://cistrome.dfci.harvard.edu/CaSNP/	Identifying CNAs in cancers and in the human genome	No	Offline
CCLE*	https://portals.broadinstitute.org/ccle	Cancer cell line encyclopedia with 36 tumor types.	Yes	Online
CGMD	http://cgmd.in/	A database of tumor genes and markers with experimental evidence	No	Offline
ChiTaRS	http://chitars.md.biu.ac.il/	Data of chimeric RNAs from eight organisms	Yes	Online
CMS	http://cbbiweb.uthscsa.edu/KMethylomes/	Gene expression and cancer methylome datasets	No	Offline
COSMIC	https://cancer.sanger.ac.uk/cosmic	Gene expression and somatic mutations in human cancer	Yes	Online
CPDB	https://www.library.ucdavis.edu/database/carcinogenic-potency-database-cpdb/	Human exposures to chemicals that cause cancer in rodent tests	No	Online
DBCAT	http://dbcat.cgm.ntu.edu.tw/	DataBase of CpG islands with a genome browser	Yes	Online
DbDEMC	https://www.picb.ac.cn/dbDEMC/	A database of differentially expressed miRNAs in human cancers	Yes	Online
dbDEPC	https://www.scbit.org/dbdepc3/index.php	A database with curated cancer proteomics data	Yes	Online
DBGC	http://bminfor.tongji.edu.cn/dbgc/index.do	Human gastric cancer-related gene expression data	No	Offline
DiseaseMeth	http://bio-bigdata.hrbmu.edu.cn/diseasemeth/	Associations between diseases and methylation of specific DNA regions	No	Online
DriverDB	http://120.110.158.132:8787/driverdbv2/cancer.php	Gene expression and driver gene/mutation identification	Yes	Online
DSTHO	http://203.199.182.73/gnsmmg/databases/dstho.html	Database of siRNAs Targeted at Human Oncogenes	No	Offline
GCOD	http://compbio.dfci.harvard.edu/gcod	Microarray data on GeneChip arrays related to human cancers	Yes	Offline
GEMiCCL	https://www.kobic.kr/GEMICCL/	Repository of cancer cell lines genotype and expression information.	Yes	Online
GeneHub-GEPIS	http://www.cgl.ucsf.edu/Research/genentech/genehub-gepis/	Human and mouse gene expression of normal and cancer tissues	Yes	Online
GSR	https://popmodels.cancercontrol.cancer.gov/gsr/	Genetic simulation resources provided by the National Cancer Institute	No	Online
HCMDB	http://hcmdb.i-sanger.com/index	Human cancer metastasis database with gene expression data	Yes	Online
HCPIN	http://nmr.cabm.rutgers.edu/hcpin	Gene expression of human cancer with protein interaction network	No	Offline
HlungDB	http://www.megabionet.org/bio/hlung	Lung cancer-related genes, proteins and miRNA with clinical data	No	Offline
HNOCDB	http://gyanxet.com/hno.html	Methylation and gene expression in Head Neck and Oral Cancer	No	Online
ISOexpresso	http://wiki.tgilab.org/ISOexpresso/main.php?cat=about	Isoform-level gene expression analysis in human cancer	Yes	Online
MCF10A	https://carcinogenome.org/MCF10A/	Visualizing data from normal and mammary tumors	No	Online
MeInfoText	http://bws.iis.sinica.edu.tw:8081/MeInfoText2/	Manual curation of gene methylation-cancer relations	Yes	Offline
MERAV	http://merav.wi.mit.edu/	Gene expression in normal tissues, cancer cell lines, and primary tumors	Yes	Online
MethHC	http://methhc.mbc.nctu.edu.tw/	Expression profiles and methylation of cancers and other diseases	No	Offline
MethmiRbase	https://madlab.cpe.ku.ac.th/TR2/?itemID=108747	Epigenetic regulated miRNA in human cancers	Yes	Online
MGDB	http://bioinfo.ahu.edu.cn:8080/Melanoma/	Multiomics catalog of genes involved in melanoma	Yes	Online
MiRCancer	http://mircancer.ecu.edu/	Expression profiles in human cancers and curated literature	Yes	Online

Name	URL	Brief description	Download of Data	Current status
MiTranscriptome	http://mitranscriptome.org/	Primary tumor, metastases, and normal or benign adjacent tissues	Yes	Online
MSGene	http://msgene.bioinfo-minzhao.org/	Comprehensive gene resource for metastasis suppressor	Yes	Online
MTCTScan	http://mulinlab.org/mtctscan	Mutations affecting cancer drug sensitivity based on genomic profiles	Yes	Online
NCI ALMANAC	https://dtp.cancer.gov/ncialmanac/initializePage.do	Information about greater antitumor activity than either agent alone	Yes	Online
NeXtProt	https://www.nextprot.org/about/nextprot	Gene expression of genes in hereditary cancers and channelopathies	Yes	Online
OncoDB.HCC	http://oncodb.hcc.ibms.sinica.edu.tw/index.htm	Quantitative trait loci of rodent models and human Hepatocellular cancer	Yes	Online
OncomiRdbB	http://tdb.ccmb.res.in/OncomiRdbB/index.htm	Data of microRNAs which are known to be deregulated in various cancers	No	Online
Oncotator	http://portals.broadinstitute.org/oncotator/	Cancer-specific annotations from public resources	Yes	Offline
Oncoyeasti	http://www.oncoyeasti.org/	Human homologs of yeast genes in samples of TCGA and CCLE	No	Online
Pedican	http://pedican.bioinfo-minzhao.org/	Gene expression in pediatric/childhood cancer	Yes	Online
PepiD	https://wukong.tongji.edu.cn/pepid	Epigenetic data in prostate cancer of human, mouse, and rat	Yes	Offline
Progenetix	https://www.progenetix.org/	Oncogenomic database that provides copy number aberration data	No	Online
S-MED	https://www.oncomir.umn.edu/SMED/basic_search.php	miRNA expression in various human sarcoma types	Yes	Online
SelTarbase	http://www.seltarbase.org/	Collection of human microsatellite instability tumor and cell line	Yes	Online
TANTIGEN	http://projects.met-hilab.org/tadb/	Tumor T cell antigens and validated epitopes for discovered peptides	No	Online
TCGA Portal*	https://www.cancer.gov/	The cancer genome atlas with multi-omic and clinical data	Yes	Online
TCRex	https://tcrex.biodatamining.be/instructions/	Predict the recognition of pathogenic & cancer epitopes by human TCR	Yes	Online
TICdb	http://203.255.191.229:8080/chimerdbv31/mindex.cdb	Gene expression and translocation breakpoints in human tumors	Yes	Offline
TUMIR	http://www.ncrnalab.com/TUMIR/	Validated resource of microRNA deregulation in various cancers	Yes	Offline
UMDTP53db	http://www.umd.be:2072/	A reference for all scientists working on p53	Yes	Online

*Databases present in the case study.
