# Supplementary material for: Fantastic databases and where to find them: Web applications for researchers in a rush
Source: Genet Mol Biol. 2021 Apr 2;44(2):e20200203. doi: 10.1590/1678-4685-GMB-2020-0203 (PMC8022358; doi:10.1590/1678-4685-GMB-2020-0203)
Supplement: Table S3 - [file 1415-4757-GMB-44-2-e20200203-s3.pdf]

Genetics and Molecular Biology
https://doi.org/10.1590/1678-4685-GMB-2020-0203

Supplementary Material to "Fantastic Databases and where to find them: Web applications for researchers in a rush"

Table S3 - Comparative databases.

Name	URL	Brief description	Download of Data	Current status
EVOG	http://neobio.cs.pusan.ac.kr/evog/	Phylogenetic evolution analysis for genes among species	No	Online
GALA	http://gala.bx.psu.edu/	Human-mouse whole-genome alignments and annotations	Yes	Online
GMO	http://gametsonline.nwsuaflmz.com/gene%20search.php	Analysis of the gametogenesis genes in human and mouse	Yes	Online
Gene Set Builder	http://www.cisreg.ca/gsb/	Gene sets and Homology annotations among species	Yes	Online
HelmCoP	http://www.nematode.net/helmcop.html	Comparative genomic data from plant, animal and human helminths	Yes	Online
Homophila	https://www.aminer.cn/pub/53e9bad0b7602d97046fde08/homophila-human-disease-gene-cognates-in-drosophila	Links between human diseases and the Drosophila genome	Yes	Online
Idiographica	http://rtools.cbrc.jp/idiographica/	Create idiograms of human, mouse, rat, and fruit fly	Yes	Online
IRView	http://ir.hgc.jp/	Interacting regions in human and mouse proteins	Yes	Online
LMPD	http://www.lipidmaps.org/	Lipidomics and genomics from several species	Yes	Online
MANTEIA	http://manteia.igbmc.fr/gene-expression-search.php	Comparative genomics of human and model organisms	Yes	Online
MDWeb	http://mmb.irbbarcelona.org/MDWeb2/	Standard molecular dynamics simulations	No	Online
Metab2MeSH	http://sartorlab.ccmb.med.umich.edu/node/5	Annotations of PubChem and Human Metabolome Database	Yes	Online
MGC	https://genecollections.nci.nih.gov/MGC/	Collection of cDNA clones for human, mouse, and rat genes	No	Online
Netview	http://netview.tigem.it/netview_project/netview_tools.html	Explore the human and mouse gene regulatory networks	Yes	Online
NHPRTR	http://nhprtr.org/resources.html	Non-human primate transcriptomes and tissue pools	Yes	Online
NRED	http://jsm-research.imb.uq.edu.au/NRED	Gene expression of long ncRNAs in human and mouse	Yes	Offline
OpenDOAR	http://v2.sherpa.ac.uk/opendoar/	Directory of open access repositories, outputs, and resources	No	Online
PhenoHM	https://phenome.cchmc.org/phenoBrowser/Phenome	Identification of genes associated with phenotypes	Yes	Online
POMO	https://ruoho.uta.fi/wp/pomo/	Omics relationships and annotations across organisms	No	Offline
POEDB	https://giladlab.uchicago.edu/orthoExon/	Orthologous exon regions in human, chimpanzee, and rhesus	No	Offline
RasMol	http://www.bernstein-plus-sons.com/software/rasmol/	Molecular graphics visualization tool	No	Online

Name	URL	Brief description	Download of Data	Current status
ReCGiP	http://klab.sjtu.edu.cn/ReCGiP/	Providing candidate genes for reproductive researches	Yes	Online
SQUAT	http://bsmc.insa-lyon.fr/squat/	Analysis tools for human, mouse, and chicken data	No	Offline
StemMapper	http://stemmapper.sysbiolab.eu/	Info of stem cell/progenitor cell types of mouse and human	Yes	Online
TiSGeD	http://bioinf.xmu.edu.cn:8080/databases/TiSGeD/index.html	Tissue-specific genes of human, mouse, and rat	No	Offline
TISSUES*	https://tissues.jensenlab.org/Search	Gene-tissue associations in human and mammalian models	Yes	Online
ToppCluster*	https://toppcluster.cchmc.org/	Comparative enrichment and network analysis of multiple gene lists of human/mammalian organisms	Yes	Online
TBrowser	http://tagc.univ-mrs.fr/tbrowser/	Human and mouse molecular interactions	Yes	Offline
tRFdb	http://genome.bioch.virginia.edu/trfdb/	Transfer RNA Fragments of human and model organisms	No	Online
Vega Browser	http://vega.archive.ensembl.org/index.html	Vertebrate genome annotation	No	Online
VISTA Browser	https://enhancer.lbl.gov/frnt_page_n.shtml	Experimentally validated human and mouse enhancers	Yes	Online

*Databases present in the case study.
