# Supplementary material for: Fantastic databases and where to find them: Web applications for researchers in a rush
Source: Genet Mol Biol. 2021 Apr 2;44(2):e20200203. doi: 10.1590/1678-4685-GMB-2020-0203 (PMC8022358; doi:10.1590/1678-4685-GMB-2020-0203)
Supplement: Table S4 - [file 1415-4757-GMB-44-2-e20200203-s4.pdf]

Genetics and Molecular Biology
https://doi.org/10.1590/1678-4685-GMB-2020-0203

Supplementary Material to "Fantastic Databases and where to find them: Web applications for researchers in a rush"

Table S4 - Disease-specific and variant-disease association.

Name	URL	Brief description	Download of Data	Current status
Globin Server	http://globin.cse.psu.edu	Experimental data and tools of human hemoglobin mutations	No	Online
AutDB	http://autism.mindspec.org/autdb/Welcome.do	Genetic variations related to autism spectrum disorders	Yes	Online
BioAfrica	https://www.krisp.org.za/tools.php	Data of Human Immunodeficiency Virus proteins and other viruses	Yes	Online
C/VD	http://www.padb.org/cvd/index.html	Compiles multi-omics data of cardiovascular-related traits	No	Online
ClinVar	https://www.ncbi.nlm.nih.gov/clinvar/	Interpretations of clinical significance of variants for reported conditions	Yes	Online
DbAARD	http://genomeinformatics.dce.edu/dbAARD/	Database of Aging and age-related disorders	Yes	Offline
DbDNV	http://goods.ibms.sinica.edu.tw/DNVs/	Data of variants of duplicated gene loci in the human reference genome	Yes	Online
DbGaP	https://www.ncbi.nlm.nih.gov/gap/	Archives and distributes the results of genotype-phenotype studies	Yes	Online
dbPEC	http://ptbdb.cs.brown.edu/dbpec/	Resource of preeclampsia phenotypes and associated genes	Yes	Offline
dbSNP	http://www.ncbi.nlm.nih.gov/SNP	Disease-causing clinical mutations as well as neutral polymorphisms	Yes	Online
dbWGFP	http://bioinfo.au.tsinghua.edu.cn/dbwgfp	Data about whole-genome SNVs and their functional predictions	Yes	Offline
DECIPHER	https://decipher.sanger.ac.uk/	Genomic variants and phenotype using Ensembl resources	No	Online
Denovo-db	http://denovo-db.gs.washington.edu/denovo-db/	Contains data about human de novo variants	Yes	Online
DES-Mutation	https://www.cbrc.kaust.edu.sa/des-mutation/mykbase/	Mutation-focused knowledge-base of Pubmed articles	No	Offline
DGP	http://maine.ebi.ac.uk:8000/services/dgp	Probability scores for genes being involved in hereditary disease	Yes	Offline
DGV	http://dgv.tcag.ca/	Collection of structural variation in the human genome	No	Online
Digital Ageing Atlas	http://ageing-map.org/	Integrated data about human ageing changes and pathologies	Yes	Online
Diseasome	http://www.kobic.kr/diseasome/	Genetic variation in disease focus on polymorphisms	No	Online
DisGeNET	https://www.disgenet.org/	Variant and gene-disease associations in more than 20000 diseases	Yes	Online
DMDM	http://bioinf.umbc.edu/dmdm/	Visualization of human coding disease-related mutations and SNPs for each protein domain	Yes	Online
EGA	https://www.ebi.ac.uk/ega/	Reference data collections for human genetics research	Yes	Online
FIDD	http://www.uwcm.ac.uk/uwcm/mg/fidd/	Frequency of inherited human disorders of over 200 conditions	No	Offline

Name	URL	Brief description	Download of Data	Current status
fitSNPs	http://fitsnps.ucsf.edu/index.php	Functionally interpolating SNPs from differential gene expression	Yes	Offline
Follicle Online	http://mcg.ustc.edu.cn/sdap1/follicle/index.php	Folliculogenesis-related experimental data from 23 model organisms, including human	No	Offline
GeneReviews	https://www.uniprot.org/database/DB-0188	Contains phenotypic information and information on selected variants	Yes	Online
Genome Trax	Genome Trax Search Tool	Variant analysis of whole genome, exome, and targeted sequences	Yes	Online
HbVar	http://globin.cse.psu.edu/hbvar/menu.html	Hemoglobin variants, hemoglobinopathies all types of thalassemia	Yes	Online
HGMD	http://www.hgmd.cf.ac.uk/ac/index.php	Compiles information related to disease-related genetic variation	No	Online
HGSVP	https://www.internationalgenome.org/human-genome-structural-variation-consortium/	Map of structural variation with genomic assays	Yes	Online
HGV&TB	http://genome.igib.res.in/hgvtb/index.html	Human genes and genetic variants associated with Tuberculosis	Yes	Online
HMDD	http://www.cuilab.cn/hmdd	Experimental evidence for human miRNA and disease associations	Yes	Online
HPO	https://hpo.jax.org/app/	Vocabulary of phenotypic abnormalities encountered in human disease	Yes	Online
Hu.MAP	http://hu.proteincomplexes.org/	A comprehensive view of protein complexes of disease genes	Yes	Online
Chr21 SNP DB	http://csnp.unige.ch/	Genetic variation of human chromosome 21 genes	Yes	Offline
HUMSAVAR	https://www.uniprot.org/docs/humsavar	An index of human polymorphisms and disease mutations	Yes	Online
InvFEST	http://invfestdb.uab.cat/	Catalog of non-redundant human polymorphic inversions	No	Online
Ion Channels Portal	https://www.nextprot.org/portals/navmut	Present phenotypes caused by genetic variations in voltage-gated sodium channels	Yes	Online
ITHANET	https://www.ithanet.eu/db/ithagenes	Archive of sequence variations affecting hemoglobin disorders	No	Online
Kaviar	http://db.systemsbiology.net/kaviar/cgi-pub/Kaviar.pl	Compilation of human SNVs collected from many and diverse sources	Yes	Online
KMeyeDB	http://mutationview.jp/MutationView/jsp/index.jsp	A database of human gene mutations that cause eye diseases	Yes	Online
LaforaDB	http://projects.tcag.ca/lafora/	A human Lafora disease (LD) mutation database	Yes	Online
LongevityMap	http://genomics.senescence.info/longevity/	A catalog of human genetic variants associated with longevity	Yes	Online
LOVD	http://www.lovd.nl/	Gene-centered collection and display of DNA variations	No	Online
LSDBs	https://grenada.lumc.nl/LSDB_list/lsdbs	Gene sequence variation associated with human phenotypes	No	Online
MARRVEL*	http://marrvel.org/	Model organism aggregated resources for rare variant exploration	Yes	Online
MelanomaMine	http://melanomamine.bioinfo.cnio.es/	Data of melanoma-related biomedical knowledge resources	Yes	Online
MitoVariome	https://www.kobic.re.kr/MitoVariome	Genetic variation in the mitochondrial genome with haplogroups	Yes	Offline
Mutalyzer	https://mutalyzer.nl/	Sequence variants according to the standard human sequence variant nomenclature (HGVS)	No	Online
MutPred	http://mutpred.mutdb.org/	Classify an amino acid substitution as disease-associated or neutral	No	Online
NetChop	http://www.cbs.dtu.dk/services/NetChop/	Neural network predictions for cleavage sites of the proteasome	No	Online
OCDB	http://alpha.dmi.unict.it/ocdb/	Data of genes, miRNAs, and drugs for obsessive-compulsive disorder	Yes	Offline

Name	URL	Brief description	Download of Data	Current status
OMIM	https://omim.org/	Online mendelian inheritance in man with genes and genetic disorders	Yes	Online
PAHKB	https://bioinfo.uth.edu/PAHKB/	Records core pulmonary hypertension (PH)-related genes	Yes	Online
PanelApp	https://panelapp.genomicsengland.co.uk/	Virtual gene panels of disorders to be created, stored and queried	Yes	Online
PaPI	http://papi.unipv.it/	Score variants according to the damage their protein-related function	No	Online
PEDB	http://www.pedb.org	Prostate gene expression database	Yes	Offline
pfSNP	http://pfs.nus.edu.sg/	Analysis of SNPs and your possible functionality from sequence motifs	Yes	Offline
PharmGKB	https://www.pharmgkb.org/	Pharmacogenomics and impact of genetic variations on drug response	Yes	Online
PhenCode	http://globin.bx.psu.edu/phencode	Human phenotype and clinical data in various locus-specific databases	No	Offline
Phenocarta	Expression Experiments	Phenotypes tree of diseases, human and mammalian species	Yes	Online
PhenoCHF	http://www.nactem.ac.uk/PhenoCHF/	Phenotypic information related to integrating heterogeneous resources	Yes	Online
PhenoDB	https://researchphenodb.net/	Exome/genome sequencing to identify the genes and variants	Yes	Online
PhenoDigm	https://www.sanger.ac.uk/science/tools/phenodigm	Phenotype comparisons for disease and gene models	Yes	Online
PhenoHM	https://phenome.cchmc.org/phenoBrowser/Phenome	Human-mouse comparative phenome-genome and orthologous	Yes	Online
Phenopedia	https://phgkb.cdc.gov/PHGKB/startPagePhenoPedia.action	Genetic association studies in order to facilitate knowledge synthesis	Yes	Online
PhenoTips	https://phenotips.com/	Data about phenotypic information for patients with genetic disorders	Yes	Online
PhosphoPOINT	http://kinase.bioinformatics.tw/	Comprehensive human kinase interactome and phospho-protein	No	Offline
PhosphOrtholog	http://www.phosphortholog.com/	Cross-species mapping of orthologous protein modifications	Yes	Online
PolyPhen2	http://genetics.bwh.harvard.edu/pph2/	Impact of an amino acid substitution on the structure and function	No	Online
PredictSNP2	http://loschmidt.chemi.muni.cz/predictsnp2/	Evaluation of the pathogenic effect of SNPs within the human genome	Yes	Online
PTM-SNP	http://gcode.kaist.ac.kr/ptmsnp	Collection of non-synonymous SNPs that affect post-translational modification sites	No	Offline
PubAngioGen	http://www.megabionet.org/aspd/	The connection between angiogenesis and diseases at multi-levels	Yes	Offline
RatMine / InterMine	http://ratmine.mcw.edu/ratmine/begin.do	Integrates many types of data for human, mice, rat, and other species	Yes	Online
RAvariome	http://www.h-invitational.jp/hinv/rav/	Data about variants of rheumatoid arthritis	No	Online
SNP2TFBS	https://ccg.epfl.ch/snp2tfbs/	Approach for identifying regulatory variants	No	Online
SNPDelScore	https://www.ncbi.nlm.nih.gov/research/snpdelscore/	Analysis of deleterious effects of noncoding variants	Yes	Online
SNPedia	https://www.snpedia.com/	Functional consequences of human genetic variation as published in studies	No	Online
SNPeffect	https://snpeffect.switchlab.org/	Information about phenotyping human single nucleotide polymorphisms	Yes	Online
SNVBox	https://karchinlab.org/apps/appSnvBox.html	Prediction of the impact of either germline or somatic SNVs	Yes	Online
TMREC	http://210.46.85.180:8080/TMREC/	Regulatory cascades in specific disease by TF, miRNA or disease name	Yes	Offline
UGAHash	http://ugahash.uni-frankfurt.de/	Accession numbers to genomic features with a focus on lncRNAs	No	Online
UMD-Predictor	http://umd-predictor.eu/index.php	Identify potential pathogenic variations, associates with omics data	Yes	Online

Name	URL	Brief description	Download of Data	Current status
VarSome*	https://varsome.com/	Variant and gene annotation, curated from public repositories	Yes	Online

*Databases present in the case study.
