# Supplementary material for: Fantastic databases and where to find them: Web applications for researchers in a rush
Source: Genet Mol Biol. 2021 Apr 2;44(2):e20200203. doi: 10.1590/1678-4685-GMB-2020-0203 (PMC8022358; doi:10.1590/1678-4685-GMB-2020-0203)
Supplement: Table S5 - [file 1415-4757-GMB-44-2-e20200203-s5.pdf]

Genetics and Molecular Biology
https://doi.org/10.1590/1678-4685-GMB-2020-0203

Supplementary Material to “Fantastic Databases and where to find them: Web applications for researchers in a rush”

Table S5 - Gene expression databases.

Name	URL	Brief description	Download of Data	Current status
AgeFactDB	http://agefactdb.jenage.de/	Integration of ageing phenotype and lifespan data	Yes	Online
aGEM	http://agem.cnb.csic.es	Anatomic gene expression mapping	Yes	Offline
Allen Brain Atlas	https://alleninstitute.org/	Analysis of human brain, the human cell and the immune system	Yes	Online
AllerGAtlas	http://biokb.ncpsb.org/AlleRGatlas/	Data of human allergy genes and their related diseases	Yes	Online
AltExtron	http://bioinformatics.org.au/tools/altExtron/	Gene expression of human and mouse transcript sequences	Yes	Online
ANGIOGENES	http://angiogenes.uni-frankfurt.de/	RNA-seq endothelial data of human, mouse and zebrafish	Yes	Online
AnnoLnc	http://annolnc.cbi.pku.edu.cn/index.jsp	A web server for systematically annotating novel human lncRNAs	Yes	Offline
arrayMap	https://arraymap.org/	Gene expression and copy number profiling data in human cancer	Yes	Online
AS-ALPS	http://as-alps.nagahama-i-bio.ac.jp/index.php	Alternative splicing-induced alteration of protein structure	Yes	Online
ASMBrainBlood	https://epigenetics.essex.ac.uk/ASMBrainBlood/	Expression profile of human brain correlated within whole blood	Yes	Online
ASpedia	http://combio.snu.ac.kr/aspedia/index.html	Splicing annotation integrating genomic information	Yes	Online
ASPicDB	https://www.cineca.it/ASPicDB/	Annotation of predicted isoforms of human genes	Yes	Offline
Cellosaurus	https://web.expasy.org/cellosaurus/	Information about immortalized cell lines, naturally immortal cell lines, finite life cell lines of many model organisms and human	Yes	Online
CHESS	http://ccb.jhu.edu/chess/	Human RNA sequencing experiments produced by the GTEx project	Yes	Online
Cistrome DB	http://dc2.cistrome.org/	ChIP-seq and DNase-seq data in mouse and human	Yes	Online
Cortecon	http://cortecon.neuralsci.org/	Captures gene expression in the developing cortex	Yes	Online
CSPA	https://wlab.ethz.ch/surfaceome/	Cell surface protein atlas	Yes	Online
CSTEA	http://comp-sysbio.org/cstea/	Cell state transition expression and time-course gene expression	Yes	Online
CYCLONET	http://cyclonet.biouml.org/	A database on cell cycle regulation in eukaryotes	No	Online
DAnCER	http://wodaklab.org/dancer/	Genes with chromatin modification of model organisms, and human	Yes	Online
DASHR	http://dashr2.lisanwanglab.org/	Database of small human noncoding RNAs	Yes	Online
dbCRID	http://c1.accurascience.com/dbCRID/	Database of chromosomal rearrangements in diseases	Yes	Online
dbOrg	https://biodbnet-abcc.ncifcrf.gov/	Provides downloadable files of organism wide conversions	Yes	Online

Name	URL	Brief description	Download of Data	Current status
DisGeNET	https://www.disgenet.org/	Variant-disease associations and gene-disease associations	Yes	Online
DRG TXome	https://bbs.utdallas.edu/painneurosciencelab/sensoryomics/drgtxome/	Gene expression profiles across orthologous human and mouse genes	Yes	Online
EPConDB	http://www.cbil.upenn.edu/node/90	Novel pancreatic genes through the sequencing and analysis	Yes	Online
EPITRANS	http://epitrans.org	Represents relationships between gene expression and epigenetic	No	Offline
ESCAPE*	http://www.maayanlab.net/ESCAPE/	A mouse and human embryonic stem cells centered integrating data	Yes	Online
ESTDAB	https://www.ebi.ac.uk/ipd/estdab/	Data of HLA type, and immunologically characterised tumour cells	No	Online
euL1db	http://eul1db.ircan.org/	Retrotransposon insertion polymorphisms in healthy or pathological human samples and published in journals.	Yes	Online
EuroDia	http://eurodia.vital-it.ch	Gene expression performed on beta-cells of human, mouse and rat	Yes	Online
ExpEdit	https://bioinformatics.cineca.it/expedit/	RNA editing user-specified sites supported by RNA-Seq experiments	Yes	Online
F-SNP	http://compbio.cs.queensu.ca/F-SNP/	Identification of disease-causing SNPs in association studies	Yes	Offline
Fantom/EdgeExpress	https://fantom.gsc.riken.jp/4/edgeexpress/view/#5558263	Gene expression and miRNA using gene-centric and sub-network	Yes	Online
FARNA	https://www.cbrc.kaust.edu.sa/farna/?page=home	ncRNA information related to expression, pathways and diseases	No	Online
FLJ	http://flj.lifesciencedb.jp/top/	mRNA variations of human genome and cDNA sequences	Yes	Online
G4LDB	http://www.g4ldb.org/ci2/index.php	G-quadruplexes in human telomeres and gene promoter regions	No	Offline
GEMiCCL	https://www.kobic.kr/GEMICCL/	Gene expression and mutations in cancer cell lines	Yes	Online
GenAge	http://genomics.senescence.info/genes/	Gene expression in ageing genes	Yes	Online
Genatlas	http://www.genatlas.org/	Gene expression with search by phenotype or ontology	Yes	Online
Gene Microglia	http://shiny.maths.usyd.edu.au/Ellis/MicrogliaPlots	Gene expression in microglia in the aged human brain	Yes	Online
GeneHub-GEPIS	http://research-public.gene.com/	Human and mouse gene expression patterns	Yes	Offline
GeneNote	https://genecards.weizmann.ac.il/cgi-bin/genenote/about_page.pl	Full-genome database of expression in healthy human tissues	No	Online
GeneTide	http://genecards.weizmann.ac.il/genetide/	Human transcriptome and annotation	No	Offline
GENT	http://medicalgenome.kribb.re.kr/GENT/	Gene expression database of normal and tumor tissues	Yes	Offline
Globin Gene	http://globin.cse.psu.edu/	Gene expression and variants in globin related genes	No	Online
GTEx*	https://gtexportal.org/home/	Gene expression with quantitative trait locus (eQTLs)	Yes	Online
H-ANGEL	http://www.jbirc.aist.go.jp/hinv/h-angel/	Human anatomic gene expression library	No	Offline
HAGR	http://genomics.senescence.info/index.php	Gene expression of human ageing using animal models and human	Yes	Online
HCMDB	http://hcmdb.i-sanger.com/index	Human cancer metastasis. Contain gene expression data	Yes	Online
Healthspan	http://functional.domains/healthspan/	Pathways and expression of health-associated gene interactions	No	Online
Hembase	http://hembase.niddk.nih.gov	Erythroblast expressed sequenced tags and erythropoiesis genes	No	Online

Name	URL	Brief description	Download of Data	Current status
HGDP	https://www.hagsc.org/hgdp/	Gene expression in different human populations	Yes	Online
HipSci	http://www.hipsci.org/	Omics in human-induced pluripotent stem cells	Yes	Online
HIVed	http://hivlatency.erc.monash.edu/	Proteins with differential expression during the human virus infections	No	Online
hmChIP	http://jilab.biostat.jhsph.edu/database/cgi-bin/hmChIP.pl	ChIP-chip and ChIPseq samples	Yes	Online
HOMER	http://bio.informatics.iupui.edu/homer	Disease ontology, gene expression, and proteomics of tissues	No	Offline
HoTResDB	http://hotresdb.bu.edu/	microarray and NGS data from non-human primates' models	Yes	Online
HPMR	http://www.receptome.org/	Transcriptome and Proteome informations	Yes	Online
HPRD	http://www.hprd.org/	Human protein reference database	No	Online
HSF	http://www.umd.be/HSF3/index.html	Human splice finder	No	Online
HuGE Navigator	https://phgkb.cdc.gov/PHGKB/hNHome.action	Genotype, phenotype, literature finder and variant mapper	No	Online
HuGEIndex	https://www.hugeindex.org/	Human gene expression index	Yes	Offline
Human Erythroblast Maturation	https://cellline.molbiol.ox.ac.uk/eryth/cgi-bin/HEM.cgi	Maturation expression patterns , comparisons, and gene queries	No	Online
IGRhCellID	http://igrcid.ibms.sinica.edu.tw/cgi-bin/index.cgi	Integrated genomic resources of human cell lines	Yes	Online
Inner Ear Transcriptome	https://www.tgen.org/patients/neurological-disorders/	Coding and non-coding transcripts of the human inner ear	Yes	Online
Intropolis	Intropolis	RNA-seq samples and exon-exon junctions	Yes	Online
Islet Regulome Browser	http://www.isletregulome.com/isletregulome/	Enhancers and transcription factor binding sites in pancreatic progenitors and adult human pancreatic islets	No	Online
Isobase	http://cb.csail.mit.edu/cb/mna/isobase/	Orthologous transcripts and functionally related proteins	Yes	Online
ISOexpresso	http://wiki.tgilab.org/ISOexpresso/main.php?cat=about	Isoform-level expression analysis in human cancer	Yes	Online
KERIS	http://igenomed.org/immune/	Expression and gene responses to inflammation among species	No	Offline
Linc2GO	http://www.bioinfo.tsinghua.edu.cn/~liuke/Linc2GO/index.html	Expression of human lincRNA	Yes	Offline
Liverbase	http://liverbase.hupo.org.cn/index2.jsp	Proteome and transcriptome of liver samples	Yes	Online
MetaQuery	http://metaquery.docpollard.org/	Quantitative analysis of specific genes in the human gut microbiome	Yes	Online
MGEx-Udb	http://resource.ibab.ac.in/MGEx-Udb/	Uterus tissues gene expression data in human and animal models	Yes	Online
MiasDB	http://47.88.84.236/Miasdb/index.php	Gene expression and alternative splicing events	Yes	Online
microRNA body	https://www.mirnabodymap.org/	RT-qPCR data and miRNA annotation in normal and diseased tissues	Yes	Online
MitoAge	http://www.mitoage.info/	Comparative analysis and gene expression of mtDNA genes	Yes	Online
MOPED	https://www.moped.proteinspire.org/	Multi-omics profiling expression in normal and disease tissues	Yes	Offline
MSGene	http://msgene.bioinfo-minzhao.org/	Metastasis suppressor gene expression	Yes	Online
NHGRI Project	https://research.nhgri.nih.gov/microarray/index.shtml	Set cDNA library clones from UniGene clusters	No	Online

Name	URL	Brief description	Download of Data	Current status
NHPRTR	http://nhprtr.org/resources.html	Transcriptome data of non-human primates	Yes	Online
NRED	http://jsm-research.imb.uq.edu.au/NRED	Gene expression of long ncRNAs in human and mouse	Yes	Offline
OncomiRdbB	http://tdb.ccmb.res.in/OncomiRdbB/index.htm	Gene and miRNA expression of cancer samples	No	Offline
ORTI	http://orti.sydney.edu.au/index.html	Transcription Factors and Target Genes (TGs)	Yes	Online
PEpiD	https://wukong.tongji.edu.cn/pepid	Prostate cancer expression and epigenetic data	Yes	Offline
PhenoScanner	http://www.phenoscanner.medschl.cam.ac.uk/	Database of human genotype-phenotype associations	Yes	Online
Primer Z	http://grch37.genepipe.ncgm.sinica.edu.tw/primerz/beginD esign.do	PCR primer design for human, mouse, or rat genes and human SNPs	Yes	Online
QMEAN	https://swissmodel.expasy.org/qmean/	Web server for protein model quality estimation	Yes	Online
REDIportal	http://srv00.recas.ba.infn.it/redidb/index.html	RNA editing and RNA-seq experiments	Yes	Online
RefEx	https://refex.dbcls.jp/index.php?lang=en	Reference gene expression for the functional analysis of genes	yes	Online
RenalDB	http://renaldb.uni-frankfurt.de/	Whole transcriptomes, and lncRNAs of nephrology studies	No	Online
Retina	http://retina.tigem.it/	Atlas of gene expression and gene co-regulation in human retina	Yes	Online
RNALocate	http://www.rna-society.org/rnalocate/	Curated RNA subcellular localization in multiple biological processes	Yes	Online
scRNASeqDB	https://bioinfo.uth.edu/scrnaseqdb/	Tools for visualization of gene expression in single cells	Yes	Online
SIEGE	http://pulm.bumc.bu.edu/siegeDB	Expression data from epithelial cells of the human intrathoracic airway	Yes	Offline
SIGNOR	https://signor.uniroma2.it/	Signalling gene expression based on published scientific literature	Yes	Online
SpermBase	http://www.spermbase.org/Search.php	Provides large and small RNAseq data, total sperm and sperm heads	Yes	Online
Symatlas	http://symatlas.gnf.org	Expression patterns for predicted genes (human and mouse)	Yes	Offline
The Matrisome	http://matrisomeproject.mit.edu/	Gene expression of extracellular matrix (ECM) proteins	Yes	Online
The Mito browser	http://mitochondria.matticklab.com/	Human mitochondrial transcriptome of multiple cell lines and tissues	Yes	Offline
The RNA Editing	http://srv00.ibbe.cnr.it/editing/	Inosinome atlas from healthy individuals	No	Offline
TiGER	http://bioinfo.wilmer.jhu.edu/tiger/	Tissue-specific gene expression and regulatory elements	Yes	Online
TiProD	http://tiprod.cbi.pku.edu.cn:8080/index.html	Human promoter sequences for which some functional features	No	Offline
TiSGeD	http://bioinf.xmu.edu.cn:8080/databases/TiSGeD/index.htm l	Tissue gene expression profiles from human, mouse and rat	No	Offline
TISSUES	https://tissues.jensenlab.org/Search	Gene-tissue associations in human and mammalian model organisms	Yes	Online
tRFdb	http://genome.bioch.virginia.edu/trfdb/	Data of single-stranded RNA derived from mature or precursor tRNA	No	Online
TRI_tool	https://www.vin.bg.ac.rs/180/tools/tfpred.php	Prediction of protein–protein Interactions in transcriptional regulation	No	Online
TSEM	https://hood-price.isbscience.org/research/tsem/	Tissue specific encyclopedia of metabolism	Yes	Online
UMD TP53	http://www.umd.be:2072/	TP53 omics database	Yes	Offline

Name	URL	Brief description	Download of Data	Current status
VirusMINT	https://mint.bio.uniroma2.it/virusmint/	Interactions between human and viral proteins	Yes	Offline
VMH	https://vmh.life/	Human metabolism and genetics, microbial metabolism, nutrition, and diseases	Yes	Online
WeGet	https://coexpression.cmbi.umcn.nl/	Mammalian genes coexpressed with human gene set of interest	Yes	Online

*Databases present in the case study.
