# Supplementary material for: Fantastic databases and where to find them: Web applications for researchers in a rush
Source: Genet Mol Biol. 2021 Apr 2;44(2):e20200203. doi: 10.1590/1678-4685-GMB-2020-0203 (PMC8022358; doi:10.1590/1678-4685-GMB-2020-0203)
Supplement: Table S6 - [file 1415-4757-GMB-44-2-e20200203-s6.pdf]

Genetics and Molecular Biology
https://doi.org/10.1590/1678-4685-GMB-2020-0203

Supplementary Material to "Fantastic Databases and where to find them: Web applications for researchers in a rush"

Table S6 - Genomic and sequence databases.

Name	URL	Brief description	Download of Data	Current status
1000Genomes	http://www.internationalgenome.org/	A deep catalogue of human genetic variation	Yes	Online
3DSNP	http://cbportal.org/3dsnp/	Analysis of human single nucleotide polymorphisms	No	Online
3Omics	https://3omics.cmdm.tw/	Transcriptomic, proteomic and metabolomic data	Yes	Online
A Catalog of Human STR Variation	https://strider.online/	A catalog of STR variation individuals from 1000Genomes	Yes	Online
A catalog of enhancers in hESCs	http://www.medical-epigenomics.org/papers/barakat2018/	Functional enhancers in human embryonic stem cells	No	Online
ABS	https://www.crg.eu/en/programme/programmes-groups/bioinformatics-and-genomics	Annotated regulatory binding sites	Yes	Online
AceView	http://www.ncbi.nlm.nih.gov/IEB/Research/Acembly	Quality-filtered cDNA from human transcriptomes	No	Online
ActiveDriverDB	https://github.com/reimandlab/ActiveDriverDB/issues	Proteogenomics for interpreting human variants	Yes	Online
Adsn	http://adsn.ddnetbio.com/	Single cell brain atlas in human Alzheimer's disease	Yes	Online
AFND	http://www.allelefrequencies.net/	Immune gene frequencies in worldwide populations	Yes	Online
Albumin binding prediction	http://albumin.althotas.com/	Predicts serum albumin binding site of a ligand	Yes	Offline
AlloFinder	http://mdl.shsmu.edu.cn/ALF/	Exogenous allosteric modulators characterization	Yes	Online
AMAZONIA	http://amazonia.transcriptome.eu/	Atlas of transcriptome with specific tissues or cells	Yes	Online
AmtDB	https://amtdb.org/	Data of ancient human mitochondrial genomes	No	Online
ANCO-GeneDB	https://bioinfo.uth.edu/ancogenedb/	Multi Omics and drug-target data with literature mining	Yes	Online
Aneurysm DB	http://www.cuilab.cn/agd	Human, mouse and rat aneurysm-gene associations	Yes	Online
AnnoLnc	http://annolnc.cbi.pku.edu.cn/index.jsp	Annotating data of novel human lncRNAs	Yes	Offline
AVPpred	http://crdd.osdd.net/servers/avppred/	Antiviral peptide prediction server	Yes	Online
BDO	http://bioportal.bioontology.org/ontologies/BDO	Bone dysplasia ontology with omics and clinical data	No	Online
BECon	https://redgar598.shinyapps.io/BECon/	Relationship between blood and brain methylation	Yes	Online
BioGPS	http://biogps.org/	Annotation of gene and protein function	Yes	Online
BrainScope	https://brainscope.lumc.nl/brainscope	Spatial and temporal human brain transcriptome	No	Online

Name	URL	Brief description	Download of Data	Current status
CADD*	https://cadd.gs.washington.edu/	Predict impact of insertion/deletions genomic variants	Yes	Online
CAGE Basic Viewer	http://fantom31p.gsc.riken.jp/cage/	Cap-analysis gene expression for human and mouse	Yes	Offline
CanGEM	http://www.cangem.org/	Microarray, CNV and genomic in cancer	No	Offline
CCB	https://cellcycle.renci.org/	Timeline of molecular events in cell cycle progression	Yes	Online
CGMD	http://cgmd.in/	Cancer annotations and molecular descriptions	No	Offline
ChIPSummitDB	http://summit.med.unideb.hu/summitdb/index.php	TF binding sites and the topological arrangements	Yes	Online
Chorogenome	hicexplorer.readthedocs.io	Hi-C data analysis from processing to visualization	No	Online
Chr7 Annotation	www.chr7.org/	Sequence, genes, and other annotations from Chr7	Yes	Online
Cichlids	http://cichlids.biosci.gatech.edu/	Evolutionary relationship of each cichlid protein	Yes	Online
CITGeneDB	http://citgenedb.yubiolab.org	Cold-Induced thermogenesis human and mouse genes	Yes	Online
CLIMA	http://bioinformatics.hsanmartino.it/clima2/	Cell line integrated molecular and Identification tool	Yes	Online
ClinicalTrials	https://clinicaltrials.gov/	Collects public and private clinical studies	No	Online
ClinVar	https://www.ncbi.nlm.nih.gov/clinvar/	Data about germline and somatic variants	Yes	Online
ComiR	http://www.benoslab.pitt.edu/comir/	Combinatorial miRNA target prediction	Yes	Online
CRUNCH	http://crunch.unibas.ch/crunch/	ChIP-seq data analysis of human and animal models	Yes	Online
cWords	http://servers.binf.ku.dk/cwords/	Human miRNA and differential expression tool	Yes	Online
dbDSM	http://bioinfo.ahu.edu.cn:8080/dbDSM/index.jsp	Database of deleterious synonymous mutation	No	Online
dbEMT	http://dbemt.bioinfo-minzhao.org/	Epithelial-mesenchymal transition related genes	Yes	Online
DBETH	www.hpppi.iicb.res.in/btox/	Genomic database for bacterial exotoxins	Yes	Online
DBGC	http://bminfor.tongji.edu.cn/dbgc/index.do	Human gastric cancer-related data resources	Yes	Offline
dbMAE	https://mae.hms.harvard.edu/	Monoallelic expression in genome-wide studies	No	Online
dbNSFP	https://sites.google.com/site/jpopgen/dbNSFP	Predict impact of non-synonymous single-nucleotide and splice-site variants	Yes	Online
Decryptor	http://decryptor.imtm.cz/	Proteome analysis for presence of point alterations	Yes	Offline
DisGeNET*	https://www.disgenet.org/	Repository of variant-disease associations	Yes	Online
DO	https://disease-ontology.org/	Ontologies, phenotypes, and related terms	Yes	Online
Doc2Hpo	https://impact2.dbmi.columbia.edu/doc2hpo/	Human Phenotype Ontology concept curation	Yes	Online
Dr.VIS	http://www.bioinfo.org/drvis	Human disease-related viral integration sites	No	Offline
DREMECELS	http://www.bioinfoindia.org/dremecels/	Base excision and mismatch repair mechanisms associated human malignancies	Yes	Offline
E-RNAi	https://www.dkfz.de/signaling/e-rnai3//	dsRNA constructs suitable for RNAi experiments	No	Online

Name	URL	Brief description	Download of Data	Current status
EBI	https://www.ebi.ac.uk/	Platform of biological sciences	Yes	Online
eDGAR	http://edgar.biocomp.unibo.it/gene_disease_db/index.html	Disease-gene associations and Relationships among genes	Yes	Online
EpiACDev	https://cotney.research.uchc.edu/data/	Epigenomic atlas of craniofacial development	Yes	Online
EpimiRBase	https://www.epimirbase.eu/	miRNA-epilepsy association from curated literature	Yes	Online
Eponine	https://www.sanger.ac.uk/about/who-we-are	Transcription start sites mammalian genome sequence	Yes	Online
ERGR	http://bioinfo.vipbg.vcu.edu/ERGR/	Published ethanol-related gene studies	No	Offline
Exome Variant Server	https://evs.gs.washington.edu/EVS/	Exome sequencing from human tissues	Yes	Online
FirstEF	http://rulai.cshl.org/tools/FirstEF/	5' terminal exon and promoter prediction program	Yes	Online
G2Cdb	http://www.genes2cognition.org	Data of synapse proteins in physiology and disease	Yes	Online
G2D	http://g2d2.ogic.ca/	Scans a region for genes related to diseases	Yes	Offline
GENCODE	https://www.gencodegenes.org/human/	Annotation for the human and mouse genomes	Yes	Online
GenePAtlas	http://biocc.hrbmu.edu.cn/GPA/	Collection of genes, miRNAs and lncRNAs perturbed by knockdown or overexpression	Yes	Online
Gene Set Builder	http://www.cisreg.ca/gsb/	Personalized sets of genes	Yes	Online
Gene Wiki	http://en.wikipedia.org/wiki/Portal:Gene_Wiki	Gathers Wikipedia articles about human genes	No	Online
GeneCards	genecards.org/	Biomedical knowledgebase of human biological data	No	Online
Geneimprint	http://www.geneimprint.com/site/home	Imprinting genes from different species and human	Yes	Online
GeneLoc	https://genecards.weizmann.ac.il/geneloc/about_new.shtml	The integrated map for human chromosomes	Yes	Online
GeneMap	www.ncbi.nlm.nih.gov/genemap	Mapping, annotations and associated gene data	Yes	Online
GeneSeeker	http://www.cmbi.ru.nl/GeneSeeker/	Candidate genes related to a genetic disorder	No	Offline
Genome Ref	https://www.ncbi.nlm.nih.gov/grc/report-an-issue	Reference genomes consortium of multiple species	Yes	Online
GUIDES	http://guides.sanjanalab.org/#/	CRISPR knockout to target human or mouse genes	Yes	Online
H-InvDB	http://www.h-invitational.jp/	Human-transcriptome and alternative splicing	Yes	Online
HADb	http://autophagy.lu/index.html	Human autophagy database	No	Online
Harmonizome*	amp.pharm.mssm.edu/Harmonizome/	Datasets about genes and proteins	Yes	Online
Harvester	http://harvester.fzk.de/harvester/	Bioinformatic data and servers for human proteins	No	Offline
HelmCoP	http://www.nematode.net/helmcop.html	Comparative genomic data from plant, animal and human helminths	Yes	Online
HEROD	http://bidd2.nus.edu.sg/herod/index.php	Human ethnic and regional specific omics	Yes	Offline
HERVd	https://herv.img.cas.cz/	Human endogenous retroviruses data	No	Online
HExpoChem	http://www.cbs.dtu.dk/services/HExpoChem-1.0/	Explore human health risk from chemical exposure	No	Online

Name	URL	Brief description	Download of Data	Current status
HGMD	http://www.hgmd.cf.ac.uk/ac/index.php	The human gene mutation database	No	Online
HGNC	https://www.genenames.org	Nomenclature for all genes of the human genome	Yes	Online
hLGDB	http://lysosome.unipg.it/	Human lysosome miRNA database	Yes	Online
HMDAD	http://www.cuilab.cn/hmdad	Human microbe-disease association	Yes	Online
HMMER3	https://www.ebi.ac.uk/Tools/hmmer/	Homologs sequences, and protein alignments	Yes	Online
HMP	https://portal.hmpdacc.org/	Human Microbiome Project	Yes	Online
HmtDB	https://www.hmtdb.uniba.it/	Mitochondrial genomes application of MtoolBox NGS	Yes	Offline
HNOCDB	http://gyanxet.com/hno.html	Head neck and oral cancer genomic data	No	Offline
HOMD	http://www.homd.org	Bacterial species present in the aerodigestive tract	Yes	Online
HomozygosityMapper	http://www.homozygositymapper.org/	A web base approach to homozygosity mapping	Yes	Online
hORFeome Database	http://horfdb.dfci.harvard.edu/	Genome-scale human ORFeome collections	No	Online
HPO	https://hpo.jax.org/app/	The human phenotype ontology disease	Yes	Online
HUMA	https://huma.rubi.ru.ac.za/	Analyze variation in protein sequence and structures	Yes	Online
Human Transporter Database	http://htd.cbi.pku.edu.cn	Molecular and genetic information of transporters for personalized medicine	No	Offline
HumanMine	http://www.intermine.org	Genes, proteins, pathways, ontology, or literature	Yes	Online
HumCFS	https://webs.iiitd.edu.in/raghava/humcfs/index.html	Curated human chromosomal fragile sites	No	Online
HydPred	http://lishuyan.lzu.edu.cn/hydpred/	Identification of protein hydroxylation sites	No	Online
HyperCLDB	http://bioinformatics.hsanmartino.it/hypercldb/	The hypertextual version of the Cell Line Database	No	Online
IDADE2	http://bass.uib.es/~jaume/IDADE2/https/index.html	Adult age estimation of human skeletal remains	Yes	Online
IGSR	https://www.internationalgenome.org/1000-genomes-project-publications	The largest open collection of human variation data	Yes	Online
iLoc-Cell	http://www.jci-bioinfo.cn/iLoc-Hum	Subcellular locations of proteins with multiple sites	No	Online
IMGT	http://www.imgt.org/	ImMunoGeneTics information system	Yes	Online
Integr8	https://www.ebi.ac.uk/integr8	Data of complete genomes and proteomes	Yes	Online
integrated gene catalog	http://meta.genomics.cn/meta/home	Reference catalog of the human gut microbiome	No	Offline
IntSplice	https://www.med.nagoya-u.ac.jp/neurogenetics/IntSplice/	Predict SNV impact in introns of the human genome	No	Online
InvFEST	http://invfestdb.uab.cat/	Non-redundant human polymorphic inversions data	No	Online
ITHANET	https://www.ithanet.eu/db/ithagenes	Variations, epidemiology, and and HbF inducers info	No	Online
Kaviar	http://db.systemsbiology.net/kaviar/cgi-pub/Kaviar.pl	Compilation of human SNVs data	Yes	Online
lncRNAtor	http://lncrnator.ewha.ac.kr/	Multi Omics of animal models and human	No	Offline

Name	URL	Brief description	Download of Data	Current status
MAHMI	http://mahmi.org/	Mechanism of action of the human microbiome	No	Online
MARome	http://196.1.114.46:8080/MARome/index	Scaffold and Matrix Attachment Regions in Genome	Yes	Online
MDWeb	http://mmb.irbbarcelona.org/MDWeb2/	Web-based platform of molecular dynamics	No	Online
MEME Suite	http://meme-suite.org/	DNA motifs, transcription factor binding sites or protein domains search tool	Yes	Online
Mendel,MD	https://mendelmd.org/	Identify disease causing variants in human exome/genome sequencing data	Yes	Offline
MetaQuery	http://metaquery.docpollard.org/	Data of specific genes in the human gut microbiome	Yes	Online
MetaRanker	http://www.cbs.dtu.dk/services/MetaRanker/	Prioritize the entire protein-coding part of the human genome based on heterogeneous user-specified data sets	Yes	Online
MGDB	http://bioinfo.ahu.edu.cn:8080/Melanoma/	Melanoma genomic and transcriptomic data	Yes	Online
microDoR	http://reprod.njmu.edu.cn/cgi-bin/microdor/index.py	Predict Human miRNA-mediated gene silencing	Yes	Online
miR2GO	http://compbio.uthsc.edu/miR2GO/home.php	Comparative analyses of human miRNA functions	Yes	Online
mLASSO-Hum	http://bioinfo.eie.polyu.edu.hk/mLASSOHumServer/index.html	Human-protein subcellular localization predictor	No	Online
MSEA	https://www.metaboanalyst.ca/	Enrichment analyses for metabolomic studies	Yes	Online
mTCTScan	http://mulinlab.org/mtctscan	Analysis of mutations affecting cancer drug sensitivity based on individual genomic profiles	Yes	Online
mtDNA-Serve	https://mtdna-server.uibk.ac.at/index.html	mtDNA NGS analysis, heteroplasmy detection, and haplogroup-based contamination deconvolution	Yes	Online
Mutalyzer	https://mutalyzer.nl/	Variant effect analysis	No	Online
MutPred	http://mutpred.mutdb.org/	Predicts molecular cause of disease	No	Online
NCBI	https://www.ncbi.nlm.nih.gov/	Supplies several online resources for biological information	Yes	Online
NetChop	http://www.cbs.dtu.dk/services/NetChop/	Cleavage site analysis of the human proteasome	No	Online
NetGene2	http://www.cbs.dtu.dk/services/NetGene2/	Predictions of splice sites	No	Online
NetNGlyc	http://www.cbs.dtu.dk/services/NetNGlyc/	Predict N-Glycosylation sites in human proteins	No	Online
Netview	http://netview.tigem.it/netview_project/netview_tools.html	Human and mouse gene regulatory networks	Yes	Online
NGD	http://www.nencki-genomics.org	Nencki Genomics Database TFBS motifs	Yes	Online
NHGRI Project	https://research.nhgri.nih.gov/microarray/index.shtml	Protocols, analysis and resources	No	Online
NIF	https://neuinfo.org/	Multi omics and neuroscience portal of Wikipedia	No	Online
OKdb	http://okdb.appliedbioinfo.net/	Function, expression pattern and regulation of genes expressed in the ovary	Yes	Online

Name	URL	Brief description	Download of Data	Current status
OMIM	https://omim.org/	Catalog of mendelian diseases	Yes	Online
OncoDB.HCC	http://oncodb.hcc.ibms.sinica.edu.tw/index.htm	Oncogenomic database for Hepatocellular Carcinoma	Yes	Online
ONTOAD	http://bioportal.bioontology.org/ontologies/ONTOAD	Text mining disease ontology	No	Online
Open Targets Platform	https://www.targetvalidation.org/	Systematic drug target identification and prioritisation	Yes	Online
OrthoDisease	http://orthodisease.sbc.su.se/cgi-bin/index.cgi	Gene ortholog between human and other species	Yes	Online
OsteoporosAtlas	http://biokb.ncpsb.org/osteoporosis/index.php	Osteoporosis-related gene database	Yes	Online
OverGeneDB	http://overgenedb.amu.edu.pl	Protein overlapping genes in Human and Mouse	Yes	Online
p53FamTaG	http://www2.ba.itb.cnr.it/p53FamTaG/	p53 family target genes	No	Offline
PanelApp	https://panelapp.genomicsengland.co.uk/	Virtual gene panels related to human disorders	Yes	Online
PaPI	http://papi.unipv.it/	Classification of variants	No	Online
PathogenFinder	https://cge.cbs.dtu.dk/services/PathogenFinder/	Prediction of a bacteria's pathogenicity in human hosts	No	Online
PDID	http://biomine.cs.vcu.edu/servers/PDID/index.php	Protein-drug interactions in the structural human proteome	Yes	Online
PDZPepInt	http://modpepint.informatik.uni-freiburg.de/PDZPepInt/Input.jsp	Predict binding peptides of PDZ domains in human, mouse, fly and worm	Yes	Online
Pedican	http://pedican.bioinfo-minzhao.org/	Pediatric cancer gene database	Yes	Online
PepPSy	http://peppsy.genouest.org/query	A gene expression-based prioritization system	Yes	Online
PGP	https://www.personalgenomes.org/	Interpretation of genomic traits in human tissues	Yes	Online
PharmGKB	https://www.pharmgkb.org/	Pharmacogenomic information database	Yes	Online
PhenoHM	https://phenome.cchmc.org/phenoBrowser/Phenome	Phenome-genome cross species identification of genes associated with orthologous phenotypes	Yes	Online
Phenolyzer	http://phenolyzer.wglab.org/	Discovering genes based on user-specific disease/phenotype terms	Yes	Online
PhenomicDB	https://en.wikipedia.org/wiki/PhenomicDB	A multi-organism phenotype-genotype database	Yes	Online
Phenomizer	http://compbio.charite.de/phenomizer/	Differential diagnosis in the field of human genetics	Yes	Online
PhenoScanner	http://www.phenoscanner.medschl.cam.ac.uk/	Database of human genotype-phenotype associations	Yes	Online
PhenoTips	https://phenotips.com/	Phenotype information of genetic disorders	Yes	Online
PhosphOrtholog	http://www.phosphortholog.com/	Orthologous protein post-translational modifications	Yes	Online
PICKLE	http://www.pickle.gr/	Direct human protein-protein interactions network	Yes	Offline
PINTA	https://securehomes.esat.kuleuven.be/~bioiuser/pinta/input.php	Genome-wide protein-protein interaction network	Yes	Offline
Piphillin	http://piphillin.secondgenome.com/	Metagenomic by Inference from Human Microbiomes	Yes	Online
PolyPhen2	http://genetics.bwh.harvard.edu/pph2/	Variant effect prediction tool	No	Online

Name	URL	Brief description	Download of Data	Current status
PolySearch2	http://polysearch.cs.ualberta.ca/index	Discovering associations between human diseases, genes, drugs, metabolites, toxins and more	Yes	Online
POMO	https://ruoho.uta.fi/wp/pomo/	Plotting Omics-associations for Multiple Organisms	No	Offline
PredictSNP2	http://loschmidt.chemi.muni.cz/predictsnp2/	Variant effect prediction tool	Yes	Online
Primer Z	http://grch37.genepipe.ncgm.sinica.edu.tw/primerz/beginDesign.do	Interface for PCR primer design	Yes	Online
PrimerStation	https://ps.cb.k.u-tokyo.ac.jp/	Multiplex genomic PCR primer design tool	Yes	Online
Progenetix	https://www.progenetix.org/	Curated oncogenomic database	No	Online
ProteomicsDB	https://www.proteomicsdb.org/proteomicsdb/#overview	Enables navigation of proteomes	No	Online
ProteoRE	http://www.proteore.org/	Proteomics and Transcriptomic Research Environment	Yes	Online
PRS	http://mrcieu.mrsoftware.org/PRS_atlas/	Polygenic burden associations in the human phenome	No	Online
pseudoMap	http://pseudomap.mbc.nctu.edu.tw/php/index.php	Information of pseudogenes	No	Offline
PubGene	https://www.pubgene.com/	Identification of siRNA mechanisms in pseudogenes	Yes	Online
QMEAN	https://swissmodel.expasy.org/qmean/	Qualitative model energy analysis	Yes	Online
QTRG	https://geneticmedicine.weill.cornell.edu/genome	Reference genome research in the Qatari population	No	Online
Quokka	http://quokka.erc.monash.edu/	Prediction of kinase family-specific phosphorylation	No	Online
R spider	http://www.bioprofiling.de/gene_list.html	KEGG and Reactome pathway analysis	No	Online
RAAR	http://www.lerner.ccf.org/cancerbio/heemers/RAAR/	Regulators of Androgen Action Resource	No	Online
RasMol	http://www.bernstein-plus-sons.com/software/rasmol/	3D structure viewer, web browser helper application	No	Online
RatMine	https://omictools.com/ratmine-tool	Omics data of human, mouse, rat and other organisms	Yes	Online
RefGene	https://omictools.com/refgene-tool	Reference genomes and annotation	Yes	Online
REGene	http://regene.bioinfo-minzhao.org/	Regeneration Gene database	Yes	Online
Repositive	https://discover.repositive.io/help/searching-for-data	Multiple repositories for the human genomic data	No	Offline
RIDDLE	http://www.functionalnet.org/riddle/	Functional network and gene set analysis	No	Online
RSSsite	https://www.itb.cnr.it/rss/	Recombination signal sequences in human and mouse	Yes	Online
Saqqaq project	http://genomebrowser.binf.ku.dk/cgi-bin/hgTracks?db=hg18&position=chrX%3A151073054-151383976&hgsid=80767_Yr7Y1ao0Y92AKta3Whb9lqcaEfLg	Provides data about the genome sequence of ancestor human	Yes	Offline
ScaPD	http://bioinfo.wilmer.jhu.edu/ScaPD/	Database for human scaffold proteins	No	Online
sciAI	https://sci.ai/	Biomedical papers, trials, and projects and interlinks research findings in a semantic graph	Yes	Online
SEGEL	http://www.chengfeng.info/smoking_database.html	Smoking Effects on Gene Expression of Lung	No	Online

Name	URL	Brief description	Download of Data	Current status
Semantic Body Browser	http://sbb.cellfinder.org/	Visualising expression profiles by means of semantically annotated illustrations	Yes	Online
SNP2TFBS	https://ccg.epfl.ch/snp2tfbs/	Variant effect prediction tool	No	Online
SNPDelScore	https://www.ncbi.nlm.nih.gov/research/snpdelscore/	Deleterious effects of noncoding variants	Yes	Online
SNPs GO	https://snps-and-go.biocomp.unibo.it/snps-and-go/index.html	Variant effect prediction tool	Yes	Online
SPEED	https://speed2.sys-bio.net/	A signaling pathway annotation enrichment analysis	Yes	Online
SpindleP	http://www.cbs.dtu.dk/services/SpindleP/	Neural networks prediction of whether a human gene is located at the meiotic spindle	Yes	Online
StemCellNet	http://stemcellnet.sysbiolab.eu/	Molecular networks in the context of stem cell biology	Yes	Online
StSNP	http://ilyinlab.org/StSNP/	Structure SNP variant effect prediction tool	No	Online
SubPhosPred	http://bioinfo.ncu.edu.cn/SubPhosPred.aspx	Phosphorylation sites in subcellular compartments	No	Online
SugarBindDB	https://sugarbind.expasy.org/	Glycan binding of human pathogen lectins and adhesins data	No	Online
SURFY	http://wlab.ethz.ch/surfaceome/	Visualize the human surface proteome (surfaceome) and load your own expression data	Yes	Online
T1Dbase	http://www.t1dbase.org	Resources for study of susceptibility of T1 Diabetes	No	Online
T2D-Db	http://t2ddb.ibab.ac.in	Pathogenesis of T2 diabetes in human, mouse and rat	Yes	Online
TCGA Portal	https://www.cancer.gov/	Multi omic cancer and clinical data	Yes	Online
UCSCBrowser	https://genome.ucsc.edu/cgi-bin/hgGateway	Multi omic web resources across species	Yes	Online
UK10K	https://www.uk10k.org/	Whole genomes in the British population	Yes	Online
UMD-Predictor	http://umd-predictor.eu/index.php	Identify potential pathogenic variations	Yes	Online
UniReD	http://bioinformatics.med.uoc.gr/unired/	Annotation and non-redundant Human or Mouse proteins from UniProtKB/Swiss-Prot	No	Online
VarSome	https://varsome.com/	Compiles human genomic variants	No	Online
VDJsolver	http://www.cbs.dtu.dk/services/VDJsolver/	Human immunoglobulin VDJ recombination analysis	No	Online
VectorBase	https://www.vectorbase.org/genomes	Vectors and other organisms related with diseases	Yes	Offline
Vega	http://vega.sanger.ac.uk	Vertebrate Genome Annotation	Yes	Online
Vega Browser	http://vega.archive.ensembl.org/index.html	Major histocompatibility complex (MHC) annotation and LCR across species	No	Online
VeryGene	http://www.verygene.com	Annotation of tissue-specific/enriched genes	No	Online
VHLdb	http://vhldb.bio.unipd.it/	Interactors and mutations of the human von Hippel-Lindau tumor suppressor protein	No	Online
XenoSite	https://swami.wustl.edu/xenosite/	Predictions about the human *in vivo* metabolism of small molecules	No	Offline

Name	URL	Brief description	Download of Data	Current status
Zikv-CDB	http://zikadb.cpqrr.fiocruz.br	Data of genes associated with ZIKV, their expression and interaction with miRNAs and snoRNAs	Yes	Online

*Databases present in the case study.
