# Supplementary material for: Fantastic databases and where to find them: Web applications for researchers in a rush
Source: Genet Mol Biol. 2021 Apr 2;44(2):e20200203. doi: 10.1590/1678-4685-GMB-2020-0203 (PMC8022358; doi:10.1590/1678-4685-GMB-2020-0203)
Supplement: Table S7 - [file 1415-4757-GMB-44-2-e20200203-s7.pdf]

Genetics and Molecular Biology
https://doi.org/10.1590/1678-4685-GMB-2020-0203

Supplementary Material to "Fantastic Databases and where to find them: Web applications for researchers in a rush"

Table S7 - LncRNA and miRNA databases.

Name	URL	Brief description	Download of Data	Current status
Antagomirbase	http://bioinfopresidencycollegekolkata.edu.in/antagomirs.html	Design antagomirs using GC content and secondary structures	No	Offline
CancerNet	http://bis.zju.edu.cn/CancerNet/	Cancer-specific miRNA and protein-protein interactions	Yes	Online
CircuitsDB	http://penelope.unito.it/circuitsdb2	miRNA/TF regulatory circuits in the human and mouse genomes	Yes	Offline
ComiR	http://www.benoslab.pitt.edu/comir/	Combinatorial miRNA target prediction	Yes	Online
CSmiRTar	http://cosbi.ee.ncku.edu.tw/CSmiRTar/	Predicted targets of human and mouse miRNAs	No	Offline
cWords	http://servers.binf.ku.dk/cwords/	Gene expression analysis and prioritization of miRNA	Yes	Online
DASHR	http://dashr2.lisanwanglab.org/	sncRNA and miRNA prediction	Yes	Online
dbDEMC	https://www.picb.ac.cn/dbDEMC/	Differentially expressed miRNAs in human cancers	Yes	Online
dbSMR	http://miracle.igib.res.in/dbSMR	Predicted and validated miRNA sites and SNPs in human genome	Yes	Offline
DES-ncRNA	https://www.cbrc.kaust.edu.sa/des_ncrna/	miRNA and lncRNAs based on literature-mining	Yes	Online
DMD	http://sbbi.unl.edu/dmd/	Published and novel miRNAs discovered in dietary resources	Yes	Offline
dPORE	http://cbrc.kaust.edu.sa/dpore	miRNA genes, SNPs, and predicted TFBSs in the promoter regions	Na	Offline
E-RNAi	https://www.dkfz.de/signaling/e-rnai3//	Designing and evaluating dsRNA constructs for RNAi experiments	No	Online
exoRBase*	http://www.exoRBase.org	circRNA, IncRNA and mRNA in human blood exosomes	Yes	Online
ExprTargetDB	http://www.scandb.org/apps/microrna/	miRNA-mediated gene regulation in the HapMap LCLs	Yes	Online
fat_deposition	http://www.integratomics-time.com/fat_deposition	Human, cattle, rat, and mice miRNA related to obesity	No	Offline
GED	http://gametsepi.nwsuaflmz.com/	DNA methylation, histone modification and miRNA regulation in gametogenesis	Yes	Online
Gene Perturbation Atlas	http://biocc.hrbmu.edu.cn/GPA/	Collection of transcriptome profiles genes, miRNAs and lncRNAs	Yes	Online
hLGDB	http://lysosome.unipg.it/	miRNA and Transcription Factors in Lysosomal Storage Diseases	Yes	Online
HLungDB	http://www.megabionet.org/bio/hlung	miRNAs and clinical information in Lung cancer	Yes	Offline
HMDD	http://www.cuilab.cn/hmdd	Experiment-supported evidence for miRNA and disease associations	Yes	Online
HumanViCe	http://gyanxet-beta.com/humanvice/	ceRNA and miRNA networks in virus infected human cells	Yes	Online
IntmiR	https://www.rgcb.res.in/intmir/	Intronic miRNAs of human and mouse genome	Yes	Online
IRBase/IRFinder	http://mimirna.centenary.org.au/irfinder/database/	Online database of intron retention and intronic miRNAs	No	Online

Name	URL	Brief description	Download of Data	Current status
Lnc2Meth	http://bio-bigdata.hrbmu.edu.cn/Lnc2Meth/	lncRNAs and associated DNA methylation in diseases and cancer	Yes	Online
LncATLAS	http://lncatlas.crg.eu/	lncRNA in human cells based on RNA-sequencing datasets	Yes	Online
lnCeDB	http://gyanxet-beta.com/lncedb/	List lncRNAs that can potentially act as ceRNAs	Yes	Online
LNCediting	http://bioinfo.life.hust.edu.cn/LNCediting/	Adenosine-to-Inosine editing sites in lncRNAs across human, rhesus, mouse, and fly	Yes	Online
LNCipedia	https://lncipedia.org/	Function of RNA editing in lncRNA and miRNA	Yes	Online
LncRBase	http://bicresources.jcbose.ac.in/zhumur/lncrbase/	Overlapping small noncoding RNAs, associated Repeat Elements, methylation, and lncRNA promoter information	Yes	Online
LncRNA2Function	http://mlg.hit.edu.cn/lncrna2function	Annotate functionally a set of human lncRNAs of interest	Yes	Offline
lncRNAMap	http://lncrnamap.mbc.nctu.edu.tw/php/	lncRNAs, miRNA, and siRNAs annotations and analysis	Yes	Offline
lncRNASNP	http://bioinfo.life.hust.edu.cn/lncRNASNP#!/	Data of SNP effects on lncRNA structure and lncRNA:miRNA binding	Yes	Online
lncRNAtor	http://lncrnator.ewha.ac.kr/	Expression profile, (binding) proteins, integrated sequence curation, evolutionary scores, and coding potential of lncRNAs	No	Online
LncRNAWiki	http://lncrna.big.ac.cn/index.php/Main_Page	Community-based curation and collection of information on lncRNAs	No	Online
lncRNome	http://genome.igib.res.in/lncRNome	Biologically oriented knowledgebase for lncRNAs in humans	No	Online
MethmiRbase	https://madlab.cpe.ku.ac.th/TR2/?itemID=108747	Experimentally verified cancer-associated miRNA target genes	Yes	Online
microDoR	http://reprod.njmu.edu.cn/cgi-bin/microdor/index.py	miRNA-mediated gene silencing: mRNA degradation or translational repression	Yes	Online
microPIR	http://www4a.biotec.or.th/micropir2	Predicted miRNA target sites within human promoter sequences	Yes	Offline
microRNA.org	http://www.microrna.org/	microRNA target predictions and expression profiles in mammals	Yes	Online
microTranspoGene	http://transpogene.tau.ac.il/	Human, mouse, zebrafish and nematode miRNAs derived from Transposable elements	Yes	Offline
miR-EdiTar	http://microrna.osumc.edu/mireditar/	Prediction of miRNA binding sites	No	Online
miR-host	http://www.integratomics-time.com/miR-host/catalog	Information of intragenic miRNAs in human, mouse, and chicken	Yes	Offline
miR2Disease	http://www.miR2Disease.org	Repository for miRNA deregulation in various human diseases	Yes	Online
miR2GO	http://compbio.uthsc.edu/miR2GO/home.php	Comparative analyses of human miRNA functions	Yes	Online
miRCancer	http://mircancer.ecu.edu/	Collection of miRNA expression profiles in various human cancers	Yes	Online
miRDB*	http://mirdb.org/	Resource for miRNA target prediction and functional annotations	Yes	Online
mirDIP	http://ophid.utoronto.ca/mirDIP/	Integrative score assigned to each unique miRNA–target interaction	Yes	Online
miREnvironment	http://cmbi.hsc.pku.edu.cn/miren	Data of miRNA characteristics have roles in miRNA–EFs interactions	Yes	Offline
miRGate	http://mirgate.bioinfo.cnio.es/miRGate/	miRNA and gene isoforms lists of human, mouse and rat	Yes	Online

Name	URL	Brief description	Download of Data	Current status
miRGen	http://carolina.imis.athena-innovation.gr/diana_tools/web/index.php?r=mirgenv3	Map of transcription factor-microRNA interactions for multiple tissues and cell-lines in human and mouse	Yes	Online
MirGeneDB	https://mirgenedb.org/	Annotation of vertebrate miRNA Genes and evolutionary analysis	Yes	Online
miRNA TF loop	https://rth.dk/resources/tfmirloop/	Regulatory network containing loop motifs for human and mouse using predicted miRNA and transcription factor binding sites	Yes	Online
miRNAMap	http://mirnamap.mbc.nctu.edu.tw/	Information of miRNAs and target sites in metazoan genomes	Yes	Online
miRPathDB	https://mpd.bioinf.uni-sb.de/	miRNA pathway regulation, gene ontologies and other categories	Yes	Online
MirSNP	http://cmbi.bjmu.edu.cn/mirsnp	Polymorphisms altering miRNA target sites, miRNA-related SNPs in GWAS and eQTLs	Yes	Online
miRStart	http://mirstart.mbc.nctu.edu.tw/	Data source of human microRNA transcription start sites	No	Offline
miRvar	http://genome.igib.res.in/mirlovd	A Comprehensive database for genomic variations in miRNAs	Yes	Online
miRWalk	http://mirwalk.umm.uni-heidelberg.de/	Predict miRNA interactions from existing miRNA-target resources	Yes	Online
PITA	https://genie.weizmann.ac.il/pubs/mir07/mir07_prediction.html	Lists of predicted microRNA targets in worm, mouse, fly and human	Yes	Online
Polymirts	http://compbio.uthsc.edu/miRSNP/	Functional impact of genetic polymorphisms in miRNA seed regions and miRNA target sites	Yes	Online
PuTmiR	https://www.isical.ac.in/~bioinfo_miu/TF-miRNA/TF-miRNA.html	Direct and indirect regulation of human miRNAs	Yes	Online
RenalDB	http://renaldb.uni-frankfurt.de/	Human, mice, zebrafish lncRNAs in nephrotic tissues and cells	No	Online
S-MED	https://www.oncomir.umn.edu/SMED/basic_search.php	miRNA expression in various human sarcoma types	Yes	Online
SlideBase	http://slidebase.binf.ku.dk/	microRNAs expressed/used in a specified set of cells/tissues	Yes	Online
The Functional lncRNA Database	http://www.valadkhanlab.org/database	A repository of mammalian lncRNA that have been experimentally shown to be both non-coding and functional	No	Online
TissueAtlas	https://ccb-web.cs.uni-saarland.de/tissueatlas/	An atlas of miRNA expression in multiple human tissues	Yes	Online
TriplexRNA	https://triplexrna.org/	Human genes or miRNAs in RNA triplexes	Yes	Online
TUMIR	http://www.ncrnalab.com/TUMIR/	Validated resource of microRNA deregulation in various cancers	Yes	Online
UCbase & miRfunc	http://microrna.osu.edu/.UCbase4	Correlation between miRNAs, ultraconserved sequences and the disorders related to their aberrant expression	Yes	Offline
UGAHash	http://ugahash.uni-frankfurt.de/	lncRNAs resources	No	Online
Vir-Mir db*	http://alk.ibms.sinica.edu.tw/cgi-bin/miRNA/miRNA.cgi	predicted viral miRNA candidate hairpins	Yes	Online

*Databases present in the case study.
