# Supplementary material for: Fantastic databases and where to find them: Web applications for researchers in a rush
Source: Genet Mol Biol. 2021 Apr 2;44(2):e20200203. doi: 10.1590/1678-4685-GMB-2020-0203 (PMC8022358; doi:10.1590/1678-4685-GMB-2020-0203)
Supplement: Table S8 - [file 1415-4757-GMB-44-2-e20200203-s8.pdf]

Genetics and Molecular Biology
https://doi.org/10.1590/1678-4685-GMB-2020-0203

Supplementary Material to "Fantastic Databases and where to find them: Web applications for researchers in a rush"

Table S8 - Proteome and protein-protein interaction databases.

Name	URL	Brief description	Download of data	Current status
2D-PAGE	http://web.mpiib-berlin.mpg.de/cgi-bin/pdbs/2d-page/extern/index.cgi	2-DPAGE maps, representing proteomes of various species	Yes	Online
AMPAD Knowledge Portal	https://www.synapse.org/#!Synapse:syn2580853/wiki/409840	Aging's Alzheimer's disease translational research	No	Online
BioCarta	http://www.biocarta.com/	Diagram library displaying gene interactions in pathways	No	Online
BRD	https://brd.nci.nih.gov/brd/	Review articles of human Biospecimen Science	No	Online
Calcium Gene Database	http://cagedb.uhlenlab.org/	Mapping genes for calcium signaling and their associated human genetic disorders	Yes	Online
CaspDB	http://caspdb.sanfordburnham.org	Potential cleavage sites in a set of all human proteins	No	Offline
CaspNeuroD	http://caspdb.sanfordburnham.org/caspneurod.php	Predicted caspase cleavage sites in human proteins associated with Neurological Diseases	No	Offline
CCSB Interactome	http://interactome.dfci.harvard.edu/	Interactomes and phenotypes in human disease	Yes	Online
ChemProt	http://potentia.cbs.dtu.dk/ChemProt/	Compilation of chemical-protein-disease annotation resources	Yes	Offline
CIDeR	http://mips.helmholtz-muenchen.de/cider/	Information from neurological and metabolic diseases	Yes	Online
ComPPI	https://comppi.linkgroup.hu/	Protein-Protein Interaction Database	Yes	Online
dbDEPC	https://www.scbit.org/dbdepc3/index.php	Curated cancer proteomics data	Yes	Online
DegraBase	http://wellslab.ucsf.edu/degrabase/	Mass spectrometry experiments in healthy and apoptotic cell lines	Yes	Offline
Degradome database	http://degradome.uniovi.es/	Protease alterations in hereditary diseases	No	Online
DenHunt	http://proline.biochem.iisc.ernet.in/DenHunt/about_database.php	Experimentally verified dengue-human interactions	Yes	Online
DEPhOsphorylation database	http://depod.bioss.uni-freiburg.de/	Data of phosphatases, their substrates, and the pathways in which they function	Yes	Online
DIIP	http://bioinfo.lab.mcgill.ca/resources/diip/	Isoform interactome prediction from experiments	Yes	Online
Dynamic Proteomics	http://www.dynamicproteomics.net/	Endogenously tagged proteins and their time-lapse microscopy movies	Yes	Offline
fat_deposition	http://www.integratomics-time.com/fat_deposition	Protein interactions in human, cattle, rat, and mice obesity	No	Offline
FixPred	http://www.fixpred.com	Protein sequences corrected by the FixPred pipeline	Yes	Offline

Name	URL	Brief description	Download of data	Current status
GutCyc	http://www.gutcyc.org/	Pathway/genome databases from the human gut	Yes	Offline
HADb	http://autophagy.lu/index.html	Autophagy related genes and proteins	No	Online
HAPPI	http://discovery.informatics.uab.edu/HAPPI/	Human annotated and predicted protein interaction	Yes	Online
Harvester	http://harvester.fzk.de/harvester/	Databases and servers for human proteins	No	Offline
HCPIN	http://nmr.cabm.rutgers.edu/hcpin	Human cancer pathway protein interaction network	No	Offline
HERVd	https://herv.img.cas.cz/	Human endogenous retroviruses database	No	Online
HGPD	https://hgpd.lifesciencedb.jp/cgi/	Human Gene and Protein Database	Yes	Online
hiPathDB	https://www.kobic.re.kr/	Curated human protein and pathway data	No	Offline
HKUPP	http://www.hkupp.org/	Human kidney and urine proteome information	No	Online
HMMER3	https://www.ebi.ac.uk/Tools/hmmer/	Homologs of protein sequences, and protein alignments	Yes	Online
HmtDB	https://www.hmtdb.uniba.it/	Database of human mitochondrial genomes	Yes	Offline
HPID	http://wilab.inha.ac.kr/hpid/webforms/intro.aspx	Protein interaction information from structural and experimental data	Yes	Online
Human Interactome Map	http://www.actrec.gov.in/	Exploring the human protein interaction network	Yes	Online
Human PPI network	https://gene.sfari.org/	Functional and network organization of proteome of Autism datasets	Yes	Online
Human Proteinpedia	http://www.humanproteinpedia.org	A community portal for sharing and integration of human protein data	Yes	Online
HumanNet	http://www.functionalnet.org/humannet/about.html	Human gene functional interaction Network	Yes	Online
HuPho	http://hupho.uniroma2.it/index.php	Information about human protein phosphatases	No	Online
HuPI	https://hupi.ircm.qc.ca/	Human Proteotheque Initiative	Yes	Online
HuRI	http://www.interactome-atlas.org/	Human reference protein interactome mapping project	Yes	Online
HydPred	http://lishuyan.lzu.edu.cn/hydpred/	protein hydroxylation sites in human inherited disease	No	Online
HypoxiaDB	http://www.hypoxiadb.com/hypoxiadb.html	Catalog of human hypoxia-regulated proteins	No	Online
iLoc-Cell	http://www.jci-bioinfo.cn/iLoc-Hum	Subcellular locations of human proteins	No	Online
Instruct	http://instruct.yulab.org/	Protein interactome networks in human and six model organisms	Yes	Online
Integr8	https://www.ebi.ac.uk/integr8	An integrated views of complete genomes and proteomes	Yes	Offline
Integrated Interactions Database	http://dcv.uhnres.utoronto.ca/iid/	Tissue-specific protein-protein interactions	Yes	Online
InterMitoBase	https://mcube.nju.edu.cn/cgi-bin/intermitobase/home.pl	High quality protein-protein interaction (PPI) data in human mitochondria	No	Offline
InWeb_IM	https://www.intomics.com/inbio/map.html#search	Provides an integrated human PPI network	Yes	Online
KEGG PATHWAY	https://www.kegg.jp/	Collection of manually drawn pathway maps	Yes	Online
KinMap	http://www.kinhub.org/kinmap/	Interactive navigation through human kinome data	Yes	Online
Kinome NetworkX	http://www.kinasenet.ca/	A systems biology-based framework to catalogue the human kinome	Yes	Online
KLIFS	https://klifs.vu-compmedchem.nl/	Kinase-Ligand Interaction Fingerprints and Structures	No	Online

Name	URL	Brief description	Download of data	Current status
Labeledln	https://ftp.ncbi.nlm.nih.gov/pub/lu/LabeledIn/	Drug indications via text-mining	No	Online
Ligandbook	https://ligandbook.org	Force-field parameters of small and drug-like molecules	Yes	Online
Membranome	https://membranome.org/	3D transmembrane domains and classification	Yes	Online
MiasDB	http://47.88.84.236/Miasdb/index.php	Interactions between human splicing factors, RNA elements, transcription factors, kinases and modified histones	Yes	Online
MitoP2	http://www.mitop2.de/	Evaluated yeast, mouse, and human mitochondrial proteins	No	Online
mLASSO-Hum	http://bioinfo.eie.polyu.edu.hk/mLASSOHumServer/index.html	Interpretable human-protein subcellular localization	No	Online
Molecular INTeraction Database	http://mint.bio.uniroma2.it/mint/	Molecular interactions by extracting experimental details from work published	Yes	Offline
NetNGlyc	http://www.cbs.dtu.dk/services/NetNGlyc/	Predict N-Glycosylation sites in human proteins	No	Online
neXtProt	https://www.nextprot.org/about/nextprot	Proteomics data for over 85% of human proteins	Yes	Online
PaPI	http://papi.unipv.it/	Predict impact of variants estimating the probability to damage their protein-related function	No	Online
PCDq	http://h-invitational.jp/hinv/pcdq/	Human protein complex database	No	Online
PDID*	http://biomine.cs.vcu.edu/servers/PDID/index.php	Molecular-level putative protein-drug interactions in the structural human proteome	Yes	Online
PepSweetener	https://glycoproteome.expasy.org/pepsweetener/app/	Manual annotation of MS data for intact glycopeptides	Yes	Online
PhenoPPIOrth	http://jjwanglab.org/PhenoPPIOrth/	Orthology and protein-protein interaction information to infer gene-phenotype associations for multiple species	Yes	Offline
PhosphoPICK	http://bioinf.scmb.uq.edu.au/phosphopick/phosphopick	Method for predicting kinase substrates using cellular context information	Yes	Online
PhosphoPredict	http://phosphopredict.erc.monash.edu/	Predict kinase-specific phosphorylation substrates and sites in the human proteome	Yes	Online
PhosphOrtholog	http://www.phosphortholog.com/	Cross-species mapping of orthologous protein post-translational modifications	Yes	Online
PICKLE	http://www.pickle.gr/Search	Predicts human protein-protein interactions network	Yes	Offline
PINdb	http://pin.mskcc.org/	Multiprotein nuclear complexes of human or yeast cells	Yes	
PINTA	https://securehomes.esat.kuleuven.be/~bioiuser/pinta/input.php	Genome-wide protein-protein interaction network for the prioritization of candidate genes	Yes	Offline
PIPs	http://www.compbio.dundee.ac.uk/www-pips/index.jsp	Protein interaction bayesian method that combines information from expression, orthology, domain co-occurrence, post-translational modifications and sub-cellular location	No	Online
PolyQ	http://lightning.med.monash.edu/polyq2/	Information of human polyQ repeat-containing proteins	Yes	Offline
PrePPI	https://bhapp.c2b2.columbia.edu/PrePPI/index.html	Predicted and experimentally determined (PPIs) for yeast and human	Yes	Online
Primate Orthologous Exon Database	https://giladlab.uchicago.edu/orthoExon/	Unique, non-overlapping, orthologous exon regions of human, chimpanzee, and rhesus macaque.	No	Offline

Name	URL	Brief description	Download of data	Current status
ProKinO	http://vulcan.cs.uga.edu/prokino/about/browser	Kinase activation and regulatory mechanisms	No	Online
Protein kinase resource	http://www.kinasenet.org/	Human kinome tree via an interactive display	No	Offline
ProteomicsDB	https://www.proteomicsdb.org/	Predicted and experimental human proteomes	Yes	Online
Quokka*	http://quokka.erc.monash.edu/	Kinase phosphorylation sites in the human proteome	No	Online
RasMol	http://www.bernstein-plus-sons.com/software/rasmol/	Protein 3D structure viewer	No	Online
ScaPD	http://bioinfo.wilmer.jhu.edu/ScaPD/	Database for human scaffold proteins	No	Online
SMPDB	https://smpdb.ca/view/SMP0000179	Small molecule pathway dataBase	No	Online
SpindleP	http://www.cbs.dtu.dk/services/SpindleP/	Location of proteins or genes in the meiotic spindle	No	Online
StSNP	http://ilyinlab.org/StSNP/	Compare protein structures, protein complexes, protein-protein interfaces and metabolic networks	No	Online
SubPhosPred	http://bioinfo.ncu.edu.cn/SubPhosPred.aspx	Phosphorylation sites in subcellular compartments	No	Online
SURFY	http://wlab.ethz.ch/surfaceome/	Visualize the human surface proteome (surfaceome)	Yes	Online
Sys-BodyFluid	http://www.biosino.org/bodyfluid/	Contains eleven kinds of body fluid proteomes info	Yes	Offline
The Proteome Browser*	http://proteomebrowser.org/tpb/home.jspx	Tissue localization, post-translational modifications, or interactions in human proteome	No	Online
TissueNet	http://netbio.bgu.ac.il/tissuenet/	Experimentally-identified PPIs with human tissues	Yes	Online
TOPPR	https://iomics.ugent.be/toppr/	The online protein processing resource	Yes	Offline
TRI_tool	https://www.vin.bg.ac.rs/180/tools/tfpred.php	Prediction of PPIs in human Transcriptional Regulation	No	Online
UniHI	http://www.unihi.org/	Analysis and visualization of human molecular interaction networks	Yes	Online
VDJsolver	http://www.cbs.dtu.dk/services/VDJsolver/	Data of human immunoglobulin VDJ recombination	No	Online
VHLdb	http://vhldb.bio.unipd.it/	Interactors and mutations of the human von Hippel-Lindau tumor suppressor protein	No	Online

*Databases present in the case study.
