# Supplementary material for: Fantastic databases and where to find them: Web applications for researchers in a rush
Source: Genet Mol Biol. 2021 Apr 2;44(2):e20200203. doi: 10.1590/1678-4685-GMB-2020-0203 (PMC8022358; doi:10.1590/1678-4685-GMB-2020-0203)
Supplement: Table S9 - [file 1415-4757-GMB-44-2-e20200203-s9.pdf]

Genetics and Molecular Biology
https://doi.org/10.1590/1678-4685-GMB-2020-0203

Supplementary Material to "Fantastic Databases and where to find them: Web applications for researchers in a rush"

Table S9 - Other databases.

Name	URL	Brief description	Download of data	Current status
AAgAtlas	http://biokb.ncpsb.org/aagatlas/	Autoantigen or autoantibody curated information	Yes	Online
CHOP CNV	http://cnv.chop.edu	Genomic Copy Number Variation (CNVs) that were derived from 2026 healthy individuals	Yes	Offline
CLIMA	http://bioinformatics.hsanmartino.it/clima2/	Molecular authentication of human cell lines	Yes	Offline
CytReD	http://www.cro-m.eu/CytReD/	Cytokine receptors, ligands, involvement in diseases	No	Online
dbMHC	https://ftp.ncbi.nlm.nih.gov/pub/mhc/mhc/Final%20Archive/	DNA and clinical data related to MHC	Yes	Online
Epipox	http://imed.med.ucm.es/epipox/	Peptides (HLA-I and HLA-II), predictions, and proteins	No	Online
G2D	http://g2d2.ogic.ca/	Precomputed candidate genes for diseases	Yes	Offline
GADGET	http://gadget.biosci.gatech.edu	Genetic traits of human phenotypic diversity	No	Online
Gene Set Builder	http://www.cisreg.ca/gsb/	Compile, store, export, and share sets of genes	Yes	Online
Genetic Association Database	https://geneticassociationdb.nih.gov/	Genetic association studies information	Yes	Online
GIANT	https://portals.broadinstitute.org/collaboration/giant/index.php/GIANT_consortium	Genetic investigation of anthropometric traits meta-analysis of genome-wide association data	Yes	Online
Global Biobank Engine	https://biobankengine.stanford.edu/	Visualize phenotypes, variants, genes, genetic correlation and human leukocyte antigens (HLA) alleles	No	Online
GuavaH	http://www.guavah.org/	Genome-wide association (GWAS) of HIV phenotypes	No	Online
GUIDES	http://guides.sanjanalab.org/#/	CRISPR knockout libraries in human or mouse genome	Yes	Online
H2P2	http://h2p2.oit.duke.edu/H2P2Home/	Cellular and infections agents in GWAS studies data	No	Online
HLA-ADR	http://www.allelefrequencies.net/hla-adr/	Exploring immunogenetic disease associations	No	Online
HLAsupE	http://www.immunoinformatics.net/HLAsupE/index.html	Human leucocyte antigens supertype-specific epitopes	Yes	Online
HomozygosityMapper	http://www.homozygositymapper.org/	Homozygous detection in SNP genotype or sequencing	Yes	Online
HPtaa database	http://www.bioinfo.org.cn/hptaa/	Human potential tumor associated antigens	No	Online
HyperCLDB	http://bioinformatics.hsanmartino.it/hypercldb/	Collection of information on human and animal cell lines	No	Online

Name	URL	Brief description	Download of data	Current status
IEDB	https://www.iedb.org/	Antibody and T-cell epitopes studied in various species	No	Online
iLoc-Cell	http://www.jci-bioinfo.cn/iLoc-Hum	Predictor for subcellular locations in proteins sites	No	Online
IMGT	http://www.imgt.org/	International immunogenetics information system	Yes	Online
IPD-IMGT/HLA	https://www.ebi.ac.uk/ipd/imgt/hla/	Provides a specialist database for sequences of HLA	No	Online
IPD-MHC	https://www.ebi.ac.uk/ipd/mhc/	Sequence repository of MHC from different species	Yes	Online
LD Hub	http://ldsc.broadinstitute.org/	Summary of GWAS studies	No	Online
MARome	http://196.1.114.46:8080/MARome/index	Annotation of scaffold and matrix attachment regions	Yes	Online
Metabolomics GWAS Server	http://metabolomics.helmholtz-muenchen.de/gwas/index.php	GWAS studies on the human metabolome	No	Online
MitoAge	http://www.mitoage.info/	Analysis of mtDNA, focus on animal longevity	Yes	Online
MNDR	http://www.rna-society.org/mndr/	Disease association and mapping disease ncRNA	Yes	Online
mtDNA-Server	https://mtdna-server.uibk.ac.at/index.html	Heteroplasmy detection and haplogroup-based mtDNA	Yes	Online
NetMHC-3.0	http://www.cbs.dtu.dk/services/NetMHC/	Human, mouse or monkey MHC Class I-peptide binding	No	Online
NetMHCIIpan	http://www.cbs.dtu.dk/services/NetMHCIIpan/	HLA-DR, HLA-DP, HLA-DQ human and mouse data	No	Online
PathogenFinder	https://cge.cbs.dtu.dk/services/PathogenFinder/	Bacterial pathogenicity estimation	No	Online
PEPVAC	http://imed.med.ucm.es/PEPVAC/	Tool of development of multi-epitope vaccines	Yes	Online
PhosphoPICK	http://bioinf.scmb.uq.edu.au/phosphopick/phosphopick	Predicting kinase substrates using cellular context info	Yes	Online
PolyDoms	https://polydoms.cchmc.org/polydoms/	Impact of variations in human genes to facilitate sequence-based association studies	No	Online
PolySearch2	http://polysearch.cs.ualberta.ca/index	Associations between human diseases, genes, drugs, metabolites, toxins and more	Yes	Online
Primer Z	http://grch37.genepipe.ncgm.sinica.edu.tw/primerz/beginDesign.do	Interface for PCR primer design	Yes	Online
PrimerStation	https://ps.cb.k.u-tokyo.ac.jp/	Multiplex genomic PCR primer design tool	Yes	Online
PRS	http://mrcieu.mrsoftware.org/PRS_atlas/	An atlas of polygenic burden associations across the human phenome	No	Online
pseudoMap	http://pseudomap.mbc.nctu.edu.tw/	Explores the relationships between TPG and its cognate gene with miRNA decoyed mechanisms	Yes	Offline
Psmir	http://bio-bigdata.hrbmu.edu.cn/Psmir/	Gene expression profiles under miRNA perturbation and drug treatment	Yes	Online
ReCGiP	http://klab.sjtu.edu.cn/ReCGiP/	Candidate genes in pigs based on bibliomics	Yes	Online
RepTar	http://bioinformatics.ekmd.huji.ac.il/reptar	Genome-wide predictions of human and mouse viral miRNAs	Yes	Offline
Semantic Body Browser*	http://sbb.cellfinder.org/	Graphically exploring an organism by means of ontologically annotated anatomical illustrations	Yes	Online
SHOGoiN	https://stemcellinformatics.org/	Human omics of ips and normal cells	No	Online

Name	URL	Brief description	Download of data	Current status
SNPDelScore	https://www.ncbi.nlm.nih.gov/research/snpdelscore/	Deleterious effects of noncoding variants using a large panel of currently available methods	Yes	Online
SNPxGE(2)	http://lambchop.ads.uga.edu/snpxge2/index.php	Human population gene expression and variation	Yes	Offline
SPIKE	https://www.cs.tau.ac.il/~spike/	Curated human signaling pathways	Yes	Online
StemCellNet	http://stemcellnet.sysbiolab.eu/	Molecular networks in the context of stem cell biology	Yes	Online
SWATHAtlas	http://www.swathatlas.org/	Immunogenetics systems biology information	Yes	Online
SysteMHC Atlas	https://systemhcatlas.org/	Atlas of immune peptidomics MS-related	No	Online
TCLP	http://celllines.tron-mainz.de/	HLA data, neo-epitopes, virus, and gene expression	Yes	Online
TCRex	https://tcrex.biodatamining.be/instructions/	Recognition of pathogenic & cancer epitopes by human T cell receptors (TCR)	Yes	Online
TFcheckpoint	http://www.tfcheckpoint.org/	Genome-scale regulatory network studies	Yes	Offline
The SBT	https://ftp.ncbi.nlm.nih.gov/pub/mhc/mhc/Final%20Archive/	MHC human data sequences	Yes	Online
VaDE	http://bmi-tokai.jp/VaDE/	Genomic polymorphisms associated to diseases, traits, and pharmacogenomics	Yes	Online
VDJdb*	https://vdjdb.cdr3.net/	Antigen-specific TCR sequences acquired by manual processing	Yes	Online

*Databases present in the case study.
